# Surveying biomolecular frustration at atomic resolution

**DOI:** 10.1038/s41467-020-19560-9

**Published:** 2020-11-23

**Authors:** Mingchen Chen, Xun Chen, Nicholas P. Schafer, Cecilia Clementi, Elizabeth A. Komives, Diego U. Ferreiro, Peter G. Wolynes

**Affiliations:** 1 Center for Theoretical Biological Physics, Rice University, Houston, TX USA; 2 Center for Theoretical Biological Physics, Department of Chemistry, Rice University, Houston, TX USA; 3 Department of Chemistry and Biochemistry, University of California at San Diego, La Jolla, CA USA; 4 Protein Physiology Laboratory, University of Buenos Aires, Buenos Aires, Argentina; 5 Department of Biosciences, Rice University, Houston, TX USA

**Keywords:** Protein folding, Computational biophysics, Drug discovery

## Abstract

To function, biomolecules require sufficient specificity of interaction as well as stability to live in the cell while still being able to move. Thermodynamic stability of only a limited number of specific structures is important so as to prevent promiscuous interactions. The individual interactions in proteins, therefore, have evolved collectively to give funneled minimally frustrated landscapes but some strategic parts of biomolecular sequences located at specific sites in the structure have been selected to be frustrated in order to allow both motion and interaction with partners. We describe a framework efficiently to quantify and localize biomolecular frustration at atomic resolution by examining the statistics of the energy changes that occur when the local environment of a site is changed. The location of patches of highly frustrated interactions correlates with key biological locations needed for physiological function. At atomic resolution, it becomes possible to extend frustration analysis to protein-ligand complexes. At this resolution one sees that drug specificity is correlated with there being a minimally frustrated binding pocket leading to a funneled binding landscape. Atomistic frustration analysis provides a route for screening for more specific compounds for drug discovery.

## Abstract (web summary)

The analysis of biomolecular frustration yielded insights into several aspects of protein behavior. Here the authors describe a framework to efficiently quantify and localize biomolecular frustration within proteins at atomic resolution, and observe that drug specificity is correlated with a minimally frustrated binding pocket leading to a funneled binding landscape.

## Introduction

**Fig. 1:**
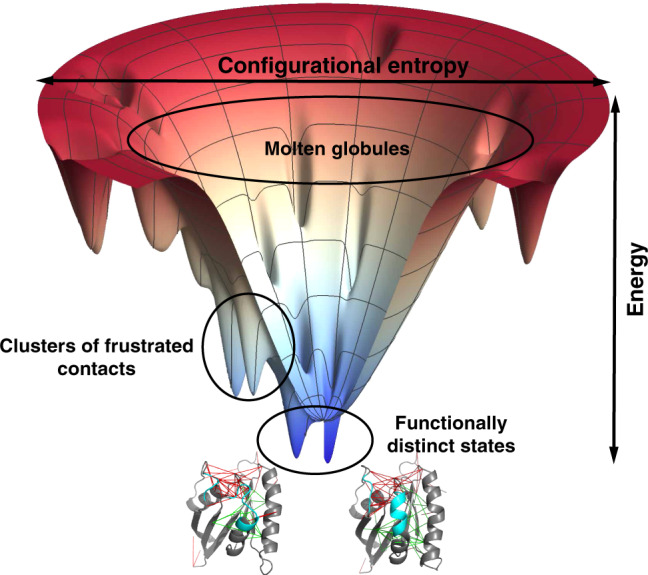
A folded biomolecule usually sits near the bottom of a funneled energy landscape. This diagram gives a sense of the statistical arrangement of states in a two-dimensional representation. The radial coordinate reflects the configurational entropy which decreases as the protein forms native contacts. The free energy of individual configurations averaged over the solvent is represented by the vertical axis. Fully denatured configurations appear at the top of the funnel. As structures form, the molecule encounters lots of barriers and local minima that may act as traps during folding. These local minima typically possess some native structure but also may make use of statistically unlikely but energetically favorable alternative non-native contacts. Such interactions are usually frustrated. If patches of frustrated contacts are localized in space they allow hinge-like motion between functionally distinct states. The frustrated contacts can take on alternative configurations and are indicated by red lines while the minimally frustrated interactions give rigidity to subdomains of the protein.

Biomolecules are beautifully sculpted mechanical devices. Evolution has achieved sculptural beauty largely by the selection of sequences of amino acids in such a way that the interactions between the amino acids of a protein in water will mutually reinforce each other most strongly in one specific structure^1^. These interactions determine the folding landscape which is then said to be “funneled”^2^. The mechanistic complexity biomolecules display is enabled also, however, by the selection of only a fraction of the residues in such a way that their interactions will conflict with each other thus compromising the stability of any single structure thereby allowing specific movements. This “frustration” of specific local interactions leads to an organized diversity of alternative states for the large biomolecule as a whole (Fig. 1). The transitions amongst these states lead to relatively constrained and well-defined functions. The analysis of biomolecular frustration using energy landscape theory^3^ has yielded insights into not only folding and misfolding, but also into protein–protein interactions^4^, allostery^5^, and enzymatic catalysis^6^.

The evolution of the patterns of frustration occurs through the repetitive, random substitution of one amino acid for another over eons of time. It is, therefore, quite natural that these patterns can usually be detected by simplifying the inter-residue interactions to a coarse-grained level in which the residues interact as rigid, nearly spherical units. Coarse-grained energy landscapes, even with their limited resolution, thus, often can do a moderately good job of predicting protein three-dimensional structure from protein sequence^1,7^. Yet, the diversity of the 20 choices of amino acids in principle allows evolution to make still finer structural distinctions possible, biasing the larger side chains to take up with higher probability some conformations rather than others. Such subtlety is not perfect, however; at equilibrium roughly 10% of the time any of the side chains can take on alternate configurations, which adds to the complexity of the functional protein-energy landscape^8–10^. Can the concept of frustration be applied at full atomic resolution?

In this paper, we quantify and explore frustration across a wide variety of protein molecules and their assemblies now at the highest level of resolution. The present approach to atomic resolution frustration analysis simplifies an earlier frustration localization algorithm working at the full atomistic level, that we recently put forward, that was computationally inefficient^11^. The greater efficiency of this new approach has enabled the large survey study we describe here. The new approach also allows us to account for the flexibility of the framework whose neglect overemphasizes repulsive steric clashes. Most of the lessons from coarse-grained frustration analysis^3^ turn out to be recapitulated at this atomic level.

The atomic level of resolution, however, also allows the extension of frustration analysis to proteins that have co-factors that typically are not amino acids and that therefore are hard to describe using coarse-grained models. The capture of co-factors by protein molecules has over evolutionary time, given biochemistry access to an extraordinary range of chemical diversity^12^. Co-factors, such as metal ions and chemically active organic ligands are essential to many enzymatic transformations and prominently figure in the energy flows of living cells. We will describe how fully atomistic frustration analysis gives some insights into how specific ligands are best captured only by specific proteins and not by others. Frustration analysis not only describes well the stability of protein–ligand association, but more importantly also puts the focus on the specificity of protein–ligand recognition. The efficient approximation made by the current calculations mimics the scenario of ligands searching for their best binding pockets by instead randomly shuffling the protein sequences to create ensembles of nonspecific binding sites and quantifying their energy landscape statistics.

Unnatural ligands are called drugs. To understand drug function then requires also an atomistic description of the binding energy landscape. Wang et al.^13^ have put forward and supported through analysis, the attractive hypothesis that the most specific and most efficacious drugs have the smoothest, most funnel-like energy landscapes for binding^14^. We show here how the present atomistic frustration analysis confirms this powerful idea showing that there is a strong correlation between there being a minimally frustrated binding pocket for a drug and the drug’s specificity, which is the sine qua non of pharmacological effectiveness.

## Results

### Spatial distribution of localized frustration in protein monomers

To study the spatial distribution of the frustrated and minimally frustrated parts of natural proteins, we first analyze a nonredundant set of 314 monomeric protein domains having high-resolution structures. For each protein, the frustration pattern can be visualized on the three-dimensional structure of the protein itself as shown in the left panels in Supplementary Fig. 1. For clarity, only the minimally frustrated interactions (green lines) and highly frustrated interactions (red lines) are drawn. The remainder of the interactions are neutral, corresponding to their being energetically near the median of the interactions in the decoy sets. In general, we see that individual domains are highly connected by minimally frustrated interactions signaling the globally funneled nature of the folding landscape. In single domains, the small minority of highly frustrated contacts are generally located at the protein surface. Clearly, local frustration is not formed randomly nor is it pervasive throughout the protein structure. For function, evolution requires a greater propensity for one part of the protein to be more frustrated than the rest of the molecule. This frustrated region can then flexibly reconfigure or “crack”^15,16^. Some functions may lead to a greater propensity for a specific region to be frustrated and thus enable specific motions. To quantify these remarks concerning the spatial distribution of the frustration indices in a statistically meaningful way, we analyze the pair distributions of local frustration indices over this monomer database.

To quantify the degree of clustering we first computed pair distribution functions between all of the contacts over the whole set (Supplementary Fig. 2b, black lines) and then separately calculated the pair distribution for contacts sorted by their frustration index. Interactions with different degrees of frustration have different patterns of spatial distribution (Supplementary Fig. 2). The neutral contacts are randomly distributed over all of the protein molecule, having a pair distribution function that follows the average protein topology (Supplementary Fig. 2b, gray and black lines). The highly frustrated contacts, in contrast, tend to be more clustered on the surfaces than expected from a random distribution (Supplementary Fig. 2b, red lines), while the minimally frustrated contacts are formed more often in the buried core. Most proteins achieve their functions through their surfaces which provide binding interfaces, that are often more flexible so as to reconfigure for accommodating a partner or for allostery. In the following sections, we will examine several different functional consequences of frustration.

### Localized frustration in allosteric proteins

**Fig. 2:**
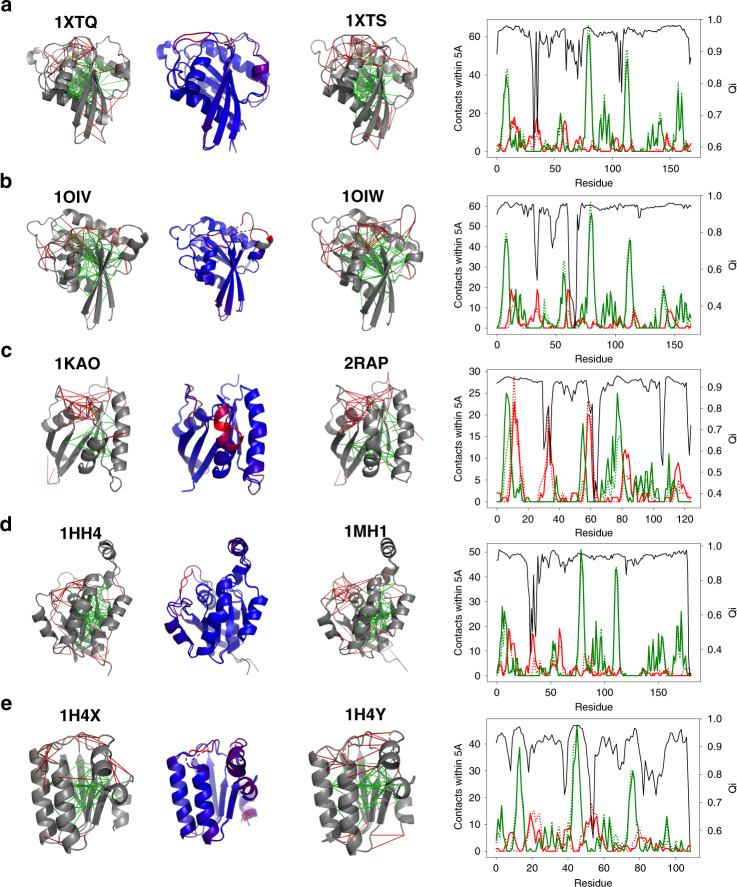
Gallery of the localized frustration and minimally frustrated networks in allosteric proteins. A structural alignment of both experimentally determined conformations is shown at the center, colored according to the structure deviation (blue low and red high). The individual conformations are shown at the sides. The protein backbone is displayed as cartoons, the interresidue interactions with solid lines. Minimally frustrated interactions are shown in green, highly frustrated interactions in red, neutral contacts are not drawn. At right, a quantification of the minimally frustrated interactions (green) or highly frustrated interactions (red) in the vicinity of each residue in **a** 1XTQ and 1XTS, **b** 1OIV, and 1OIW, **c** 1KAO and 2RAP, **d** 1HH4 and 1MH1, and **e** 1H4X and 1H4Y. The local Qi of each residue is shown in black. The quantifications of the interactions in the two configurations are shown in solid lines and dashed lines separately. We can see that the two patterns are strongly correlated with most minimally frustrated regions being nearly the same in both and the location of the frustrated regions typically only shifting by a few residues or becoming minimally frustrated.

To study allostery, we focus on the curated database of pairs of homologous proteins whose structures have been deposited for at least two globally different conformational states by Ferreiro et al.^5^. Examples of frustration patterns and allosterism are shown in Fig. 2. Visual inspection suggests that the regions that undergo reconfiguration are enriched in patches of highly frustrated interactions (Fig. 2). In order to quantify the correlation between conformational substates and local frustration, we adopted the “local-Q” scoring parameter to locate where motions have taken place between protein pairs, as introduced in Ferreiro et al.^5^. To quantify the correlation between displaced residues and their frustration levels, we compute the pair-distribution function between residues classified by their displacement and the contacts classified into different frustration classes (Supplementary Fig. 3). The regions with mobile residues are enriched in highly frustrated interactions up to 5 Å from the *C*_*α*_ of the mobile residues. The set of immobile residues is more enriched in minimally frustrated interactions. The immobile residues are more strongly correlated at long-distances through chains of minimally frustrated interactions than the contact network is as a whole, paralleling the observations of frustration patterns in allosteric proteins using the coarse-grained AWSEM-frustratometer by Ferreiro et al.^5^.

As in the monomer database, most of the interactions even in allosteric proteins are not frustrated, but evolution has made use of frustration only in localized parts of the proteins so as to allow competing low-free-energy structures which can then become differentially favored under differing thermodynamic conditions or when co-factors bind. In agreement with the survey conducted using AWSEM, the atomistic frustration analyses confirm the importance of localized frustration in protein function and evolution. Confirming this observation at the atomistic level is especially interesting because the all-atom force fields seemingly were built up from molecular inputs that explicitly do not highlight protein evolution, while the AWSEM landscape used the funnel concept in its construction by using parameter optimization strategies from landscape theory.

### Localized frustration around catalytic sites in enzymes

**Fig. 3:**
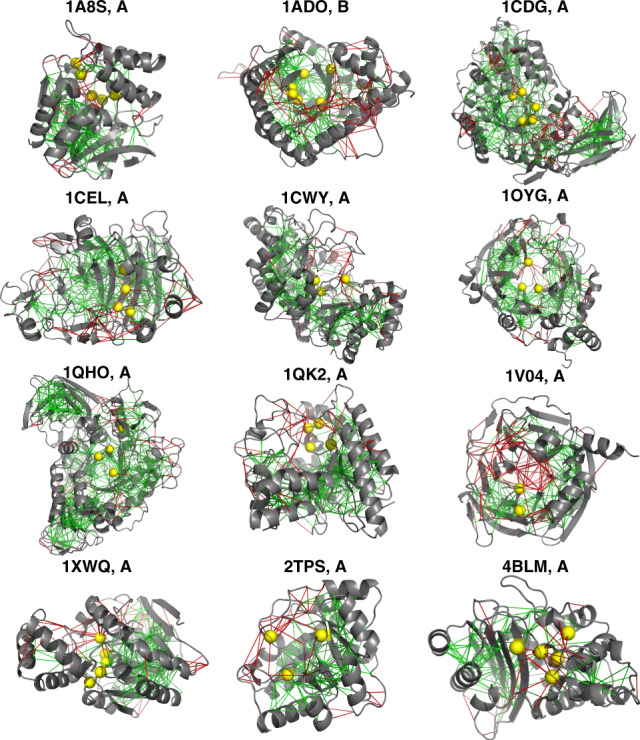
Gallery of the localized frustration and highly frustrated networks in enzymatic proteins. The protein backbone is displayed as cartoons, the interresidue interactions with solid lines. Minimally frustrated interactions are shown in green, highly frustrated interactions in red, neutral contacts are not drawn. The catalytic sites are identified from the database and shown in yellow spheres.

To analyze the local frustration distribution in enzymes, we collected all entries in the Catalytic Site Atlas for which one can find at least one high-resolution structure and for which the catalytic residues have been experimentally assigned (907 nonredundant entries). We then calculated the local frustration patterns using the atomistic frustratometer. Figure 3 shows some examples of the local frustration patterns in enzymes. It is apparent that as for other proteins, at atomic resolution the macromolecular frameworks of enzymes again are strongly interconnected by minimally frustrated interactions and that, in contrast, again the highly frustrated interactions typically form clusters. Many of the highly frustrated interactions still are at the surface of the globules, perhaps reflecting binding or allosteric sites, in agreement with the observations of general local frustration patterns from monomer proteins.

We also quantified the local frustration patterns and computed the pair distribution functions for the various classes of contacts as a function of distance (*g*(*r*)) from the catalytic sites to the center of mass of the interactions. The *g*(*r*) for each frustration class is compared with the *g*(*r*) for all contacts, which accounts for the geometry of the interaction network determined by the protein topology. As shown in Supplementary Fig. 4b, the distribution of interactions around the catalytic sites displays two characteristic peaks (black lines in Supplementary Fig. 4b: one smaller peak located around 1–2 Å, corresponding to those interactions involving the catalytic residues themselves (first shell), and a second peak between 2.5 and 5 Å (second shell). The highly frustrated interactions are more clustered around the catalytic sites, suggesting potential functional outcomes. The density of minimally frustrated interactions is less enriched in both shells around the catalytic centers. This is consistent with the observation from in vitro studies that changes in the second shell are generally needed for optimal enzyme activity^17^.

### Localized frustration at binding interfaces

In monomeric proteins, interactions among the surface residues, usually are considered as contributing less to the folding landscape than do the hydrophobic interactions of the buried residues in the core of globular proteins. The enriched frustration on the exposed residues forms functional patches. Some of the surface residues are specific for protein–protein interaction which become less frustrated once formed. We analyze the distribution of frustration indices for binding pairs in a database of protein assemblies.

The frustration patterns in the individual binding partners from those assemblies were examined for their correlation with the specific binding sites. Supplementary Figure 5 shows some examples of calculated frustration using both the atomistic frustratometer and the AWSEM frustratometer for unbound monomers. In both sets of calculations, there are highly frustrated interactions near the surfaces and their patterns, especially in the atomistic analyses, are not immediately clear. We then contrast the correlation between frustration near binding sites and frustration near the surface residues that are not involved in binding. The coarse-grained frustratometer (AWSEM) indicates clearly, that the binding sites are more enriched in highly frustrated interactions compared to non-binding residues at the surfaces. This is consistent with the idea that specific binding sites contribute to the specificity of protein–protein associations.

Landscape theory has been powerful in detailing mechanism of protein–protein association^18–20^, and the role of long-range contacts that are mediated by water on protein surfaces has been shown to favor folding by Papoian et al.^21^. For both frustratometers, those interactions that occur across water molecules, do possess the lowest fraction of minimally frustrated interactions compared with the interactions that are not mediated by water. The AWSEM frustratometer, however, does show around 24% of the water-mediated interactions display minimally frustration suggesting they provide considerable help in folding. On the other hand, the atomistic frustratometer shows only a much lower fraction of minimally frustrated water-mediated interactions and a higher level of high frustration in the water-mediated contacts than does the AWSEM. We believe this reflects a weakness of the present Rosetta force field when used without including explicit waters structurally bound to the proteins.

**Fig. 4:**
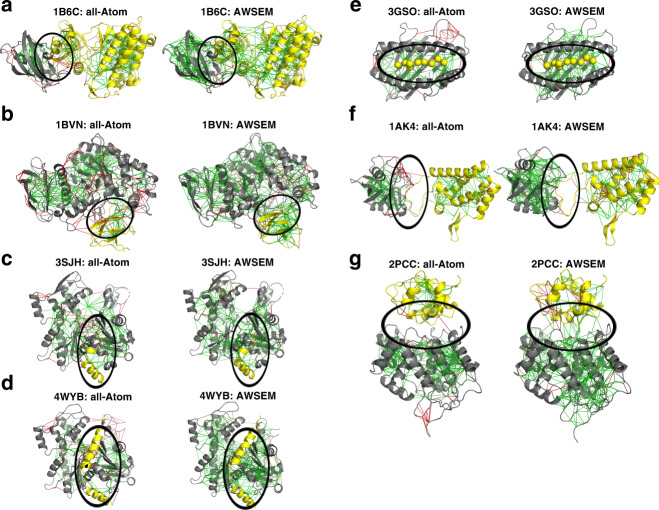
Examples of localized frustration patterns in protein complexes. For each binding complex, the frustration indices are shown as calculated for the complex using both all-atom frustration (left panel) and coarse-grained frustration (right panel). Binding interfaces in (**a**–**e**) are largely dry interfaces dominated by non-water-mediated contacts, while the interfaces in (**f**) and (**g**) are rich in water-mediated contacts.

To understand how the local frustration distribution changes upon association, we also computed and compared the frustration indices for proteins in the complexes (examples shown in Fig. 4) with those for the unbound states. For the cases when the binding interface in the complex is “dry” (Fig. 4a–e), the contacts between the protein monomers do turn out to be largely minimally frustrated according to the Rosetta force field. Figure 4c shows the frustratograms when G-actin tightly binds to *β*-Thymosin, which is an actin-binding peptide that regulates the self-assembly of actin. The frustration indices for the interdomain contacts are minimally frustrated. In contrast, when the binding interfaces in the complex are “wet” (Fig. 4f, g), the atomistic frustratometer regards the interface that is formed as being largely neutrally frustrated and as not contributing to specificity or sometimes even as being highly frustrated (note the high frustration between the two monomers in the complex 2PCC, circled regions). This indicates the waters in the interface not used in the energetic analysis are crucial to the association process.

The statistics of the frustration indices of the contacts in the interfaces reveal that short-range and long-range contacts that are not water-mediated do display similar fractions of minimally frustrating interactions in both frustratometers, but that overall in the atomistic version, the level of minimal frustration of the interface (14.2%) is much lower than what is seen when using the AWSEM potential (26.0%), which explicitly considers the role of water-mediated interactions in the model (Supplementary Fig. 8). Apparently, modeling water-mediated interactions at the atomic level for “wet” interfaces requires adding explicit waters to the Rosetta modeling or perhaps accounting for their effect using the AWSEM strategy, in some way.

### Localizing frustration in protein-molecular ligand complexes

One major advantage of employing atomistic models rather than using coarse-grained models is the fully detailed models allow the explicit incorporation of ligands or cofactors which come in a large molecular variety. We therefore now explore the use of the atomistic frustratometer to study the binding of ligands (see Supplementary Methods). During the process of a ligand binding to its receptor, different intermediate binding states emerge which have different structures with corresponding different binding energies coming from different sets of spatial contact interactions between the ligand and parts of the receptor protein. Randomly shuffling the sequence over the fixed protein backbones while allowing perturbations on the ligands imitates in a statistical sense the process of randomly docking the ligand to random protein pockets through which the ligand must pass to reach its target.

Many proteins can fold even in their apo forms without the ligand being present to structures that are not significant different from their final holo forms. To elucidate the energy landscape for ligand association to binding pockets in proteins, we now can compute and compare the frustration patterns of proteins both with and without their bound ligands using a database of more than 700 proteins in complex with their ligands. As shown in Supplementary Fig. 5, the binding pockets turn out to be largely frustrated in their apo forms, but the association with their ligands significantly increases the density of minimally frustrated interactions in the pocket. Consider the case of G-actin in complex with ATP, Latrunculin A, and Magnesium (shown in Supplementary Fig. 5a), the association of all three ligands significantly reduces the frustration level and creates a minimally frustrated environment/microenvironment for the ligands. Likewise, binding ADP strongly reduces the frustration level of its target pocket in phosphodiesterase (Supplementary Fig. 5f). The same trend continues to be meaningful when quantifying the role of frustration in the drug pockets over the full database (Supplementary Fig. 9). It is noteworthy that the enrichment of frustration around internal binding sites found in the apo forms is similar to that seen for surface residues from the monomer database.

### Correlation between ligand binding affinity and frustration level

The atomistic frustration computed through randomly shuffling the protein sequence to reproduce the molten globule states resembles evaluating the specificity of a ligand binding to a given pocket. The CO–myoglobin binding energy landscape possess a funneled shape toward the native binding basins, and the non-native states encountered during binding display nearly a gaussian distribution^22^. Frustration indices of contacts on ligand binding sites can be regarded as measuring the specificity of the interactions between the ligand and its receptor. Binding specificity compares the measured thermodynamic affinity values for different partners. The affinity of a specific partner correlates with specificity, but specificity and affinity are not the same: some ligands bind promiscuously to many partners.

**Fig. 5:**
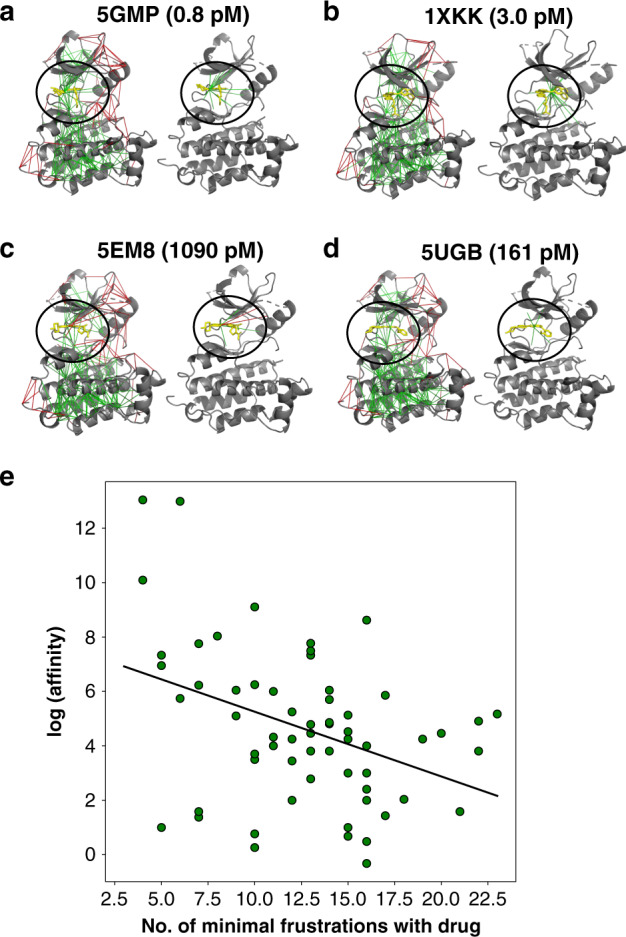
Examples of localized frustration patterns in EGFR-inhibitor complexes. For each binding complex in (**a**–**d**), the frustration indices are shown as calculated and shown on the left panel, and frustrations around the ligands only are shown on the right panel. **e** The correlation between the number of minimally frustrated interactions in each EGFR-inhibitor complex and the logarithm of binding affinity in the picomolar unit at a base of 2 is shown. A regression line is plotted to illustrated the trends with a modest pearson correlation of 0.45.

We illustrate the correlation between binding affinity and frustration level, using the atomistic frustration algorithm using a database of EGFR-kinases in complex with their inhibitors. As shown in Fig. 5, binding different inhibitors induces different changes in the frustrated patterns in the binding pocket: a strong inhibitor XTF-262 (PDB ID: 5GMP) forms more than ten minimally frustrating interactions with its pocket (Fig. 5a), while a weaker binder 5Q4 forms only three minimally frustrated interactions and once bound even brings along with it the formation of additional highly frustrated interactions in the pocket. This is consistent with the fact that 5Q4 is not an ideal binder in this pocket (Fig. 5c). Figure 5e illustrates the correlation between the frustration level and measured affinities.

### Ligand binding specificity correlates with minimal frustration

Highly efficient and specific biomolecular recognition requires both high affinity and discrimination in binding. The thermodynamic stability of a particular complex is determined by the affinity, but specificity requires disfavoring binding to other competitive biomolecules. We can see this is conceptually close to the frustration concept. Many drug design strategies have been developed that seek only to optimize the stability based on a combination of energetics and shape complementarity without explicitly considering binding specificity. This leads to drugs that bind promiscuously to several targets. While very stable binding favors achieving high specificity, it does not guarantee that the molecule will not bind strongly with other partners which are critical for a variety of physiological functions, leading to side effects.

The concepts of energy landscape theory and its underlying principle of minimal frustration have led to a good understanding of how proteins fold specifically into a tertiary configuration. Much as for protein folding, the binding process of a highly specific molecular ligand to a protein can be physically quantified and visualized as taking place largely on a funnel-like energy landscape leading toward the proper binding pocket encountering local energetic roughness along the binding paths. The extension of energy landscape theory to ligand binding by simulating the competitive states and quantifying the competitiveness of different pockets (frustration indices) thus provides a way to quantify and predict ligand–receptor binding specificity. Frustration level describes the discrimination of the native binding sites from alternative non-native or decoy binding sites. Frustration analyses using the present statistical tools naturally provides a quantitative measure of intrinsic specificity without exploring all of the receptor–ligand universe.

**Fig. 6:**
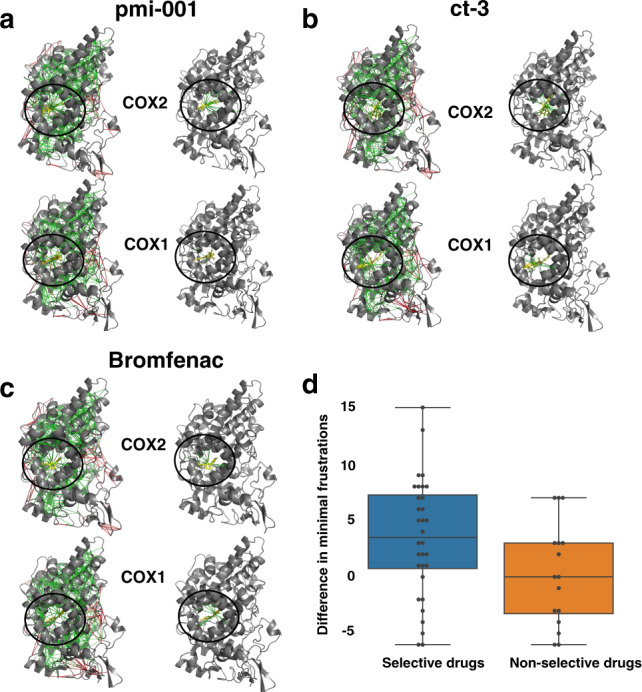
Examples of localized frustration patterns in cox-inhibitor complexes. For each drug, the frustration indices are shown as calculated and shown on the left panel, and frustrations around the ligands only are shown on the right panel. pmi-001 (**a**) is a selective inhibitor for COX2. ct-3 (**b**) and bromfenac (**c**) are inhibitors without selectivity. **d** Box plot of the increased minimally frustrated interactions in COX2 over COX1 is shown for both the selective drugs of COX2 and non-selective drugs.

To illustrate this idea, the enzyme cyclooxygenase-2 (COX-2) was chosen for local atomistic frustration analysis. Wang et al.^13^ have studied this pharmacologically important example earlier^14^. This enzyme is the target of nonsteroidal anti-inflammatory drugs that reduce fever and inflammation, such as the commonly taken drugs aspirin, motrin, telenoid, and advil. Virtual screening for COX-2 has been challenging. Along with the importance of discriminating the drugs from the diversity set of the pocket in COX-2, it is important to distinguish selective and nonselective also to its isoenzyme (COX-1). Selective drugs are more able to inhibit COX-2 than do non-selective drugs that also inhibit COX-1. We computed and compared the atomistic frustratograms of 54 COX-inhibitors in complex with both COX-1 and COX-2. Among the 54 inhibitors, 35 are known to be selective inhibitors for COX-2 and 19 are non-selective inhibitors. As shown in Fig. 6a, pmi-001 is a selective inhibitor for COX-2. Binding pmi-001 to COX-2 strongly minimizes the local frustration in the pocket. In contrast, binding pmi-001 to COX-1 only slightly changes the frustration level in the COX-1 binding pocket. In Fig. 6c, we see another inhibitor bromfenac displays similar local frustration patterns for both COX-1 and COX2. This observation is in harmony with the fact that bromfenac is a non-selective inhibitor. On average, the selective drugs display 3.5 more minimally frustrated interactions when they bind to COX-2 than when they associate with COX-1 (blue bars in Fig. 6d). The frustration patterns of the non-selective drugs in complex with both COX-1 and COX-2 do not obviously distinguish between the two enzymes (orange bars in Fig. 6d).

Those results suggest that atomistic frustration analyses of drug-protein complexes can provide a very rapid way to assess pharmacological specificity issues. Wang et al.^13^ have developed another framework to compute specificity scores of drugs by generating ensembles of docked complexes at various binding sites on the protein and calculating the *Z*-scores of the docking energy of the native pose against this ensemble. Their algorithm also indicated a strong correlation of landscape funneled-ness with specificity. The present frustration algorithm efficiently implements the same idea and also gives detailed networks of frustration which might provide hints for future drug search and optimization.

## Discussion

The interplay between water and proteins has long been recognized as critical in determining the structure and dynamics of biological macromolecules^23^. The various roles of water include the well known “hydrophobic force” and the important screening of Coulombic interactions provided by water with its high dielectric constant. Not all the effects of the water environment can be captured while treating it as a continuum. Structural waters are tightly incorporated into the protein framework and often must be regarded as part of the protein structure. Also, water molecules sometimes reside transiently near a protein surface, where they exhibit dynamics intermediate between the nearly frozen structural waters and bulk water. Recognizing this complexity, the AWSEM force field has used the energy landscape theory of protein folding to derive several sets of direct and water-mediated contact potentials for protein native interface recognition^21^. Using energy landscape analysis, Papoian et al. concluded that some of the water-mediated interactions are actually quite specific in character, facilitating biomolecular recognition through interfaces.

The Rosetta model addresses the solvation effects of waters through isotropic and anisotropic desolvation terms, where the isotropic term is supposed to cover bulk desolvation effects while an anisotropic term is intended to treat specific waters near to polar residues. Despite the incorporation of those terms, the present instantiation of Rosetta that we used seems not to capture the effects of residues interacting through the water molecule network. As a result, there is no possibility for a stabilizing interaction if two polar residues are separated by the distance of the size of one water molecule, which is the main physical–chemical effect captured by the water-mediated interaction terms in AWSEM. At present then, the water-mediated interactions as described in Rosetta tend to disfavor overall protein folding, and do not completely treat many protein–protein association processes that lead to wet interfaces.

In connection with the observation of the frustration patterns in protein–protein interfaces, dry interfaces whose interactions are made largely through direct contacts that are not mediated by water molecules do display similar frustration patterns at the atomic level as those computed through AWSEM. The wet interfaces with a large number of water-mediated interactions are less minimally frustrated with Rosetta than they turn out to be for AWSEM which considers water-mediated interactions in an explicit way. This difference is in harmony with what is seen comparing prediction accuracy using predicted models using both Rosetta and AWSEM. AWSEM outperforms Rosetta in the structural accuracy of water-mediated contacts while being somewhat less accurate in predicting direct contacts.

The current atomistic representation of biomolecules from Rosetta has many strong features but we look forward to, the missing feature of water-mediated interactions being addressed by adding in additional terms as AWSEM does. Coupling both the all-atom accuracy with coarse-grained contact potentials derived from energy landscape theory for protein folding becomes a hopeful future direction in structure prediction of complexes. In drug screening, the competitive targets that may result in side effects of the drug usually are not known. It is presently impractical to experimentally evaluate binding toward all possible competing targets, and structures of potential competitive targets are not generally available for computational evaluation of thermodynamic affinity. By taking the statistical stance from energy landscape theory, frustration analysis provides a practical way to infer binding specificity, and may provide guidance for improved molecular designs that will enhance specificity without compromising affinity. In parallel with Wang et al.^13^ who evaluated specificity through docking to random positions on a protein surface, the present approach provides a way to evaluate specificity by approximating the association into various pockets. Both approaches, under the framework of energy landscape theory, provide theoretical quantification through energetic *Z*-scores to infer specificities generically without knowing the specific alternate binding partners. In actual drug discovery, it’s usually helpful to use chemical genetics in combination with free energy perturbation methods to find better drugs starting from a lead compound. This man-made evolution of the ligands in drug design is thus often based on the binding free energies alone, but often neglects the importance of specificity. The predictive power of the atomistic frustratometer should enable better screening of ligands when employed along with traditional free energy perturbation analysis. The current atomistic frustratometer provides a framework to quantify frustration of any biomolecular systems using the Rosetta force field. This methodology can in principle be quickly adapted to any other atomistic force fields of interest. Frustration analysis certainly can profit from future vigorous forcefield development.

## Methods

### Energy landscape and frustration of real proteins

The smooth funnel-like landscape of proteins, traversed (shown in Fig. 1) as they fold, arises because many minimally frustrated interactions act together in the native structure but add incoherently in nonnative trap states. The native-like interactions are stronger than random ones and thus are most populated during folding. Energy landscape theory quantifies this idea by comparing a protein’s folding temperature (*T*_*f*_, quantifying the energy gap between folded states and traps, *σ*(*E*) = *E*_*f*_ − *E*_*g*_) to its glass transition temperature (*T*_*g*_, quantifying the roughness). If *T*_*f*_ exceeds *T*_*g*_, a globally random trap will be unstable at a temperature where the protein can still fold to its dominant folded structure. Along with the statistics of the landscape, *T*_*f*_ and *T*_*g*_ depend on the configurational entropy of the unfolded protein (Fig. 1). *T*_*f*_ depends on the gap in energies between the native structure and the average misfolded structure, while *T*_*g*_ depends on the variance of the energies of misfolded structures. The *T*_*f*_/*T*_*g*_ ratio thus is proportional to the *Z*-score of folded structure energy versus the energies of an ensemble of randomized structures. While *T*_*f*_/*T*_*g*_ is a global quantity, the corresponding *Z*-score ratio can also be computed for localized regions of a protein in the context of the protein structure. Frustration analysis takes this idea to the limit of individual interactions. While this limit is extreme, we have shown that localized frustration analysis with coarse-grained energy functions at this level of resolution correlates well with the existence of alternative states on the protein folding landscape.

### Definition of local frustration

To quantify frustration locally and especially to locate where the most frustrated interactions occur, we gather statistics about the local energy changes that occur when we systematically but locally perturb the protein sequence. The energetic changes are evaluated at specific locations in the protein structures according to the input forcefield models. Strictly speaking, these should be free energy changes averaged over the solvent degrees of freedom. The amino acids that form a particular contact in the native structure are virtually mutated to other amino acids generating a set of decoys for which the total energy of the protein is then recomputed. Sequence space is randomly sampled according to the native amino acid frequency distribution for the particular protein under consideration, giving 1000 appropriately distributed decoys for each contact. A histogram of the energy of the decoys is constructed to compare the distribution with the native energy, *E*_0_. The frustration index for the contact between the amino acids *i*, *j* is defined as the *Z*-score of the energy of the native pair compared with the N decoys:

1 $${F}_{ij\,}^{o}=({E}_{ij\,}^{0}-{E}_{{i}^{\prime}{j}^{\prime}}^{U})/\left.\sigma ({E}_{{i}^{\prime}{j}^{\prime}}^{U})\right.,$$

where ${E}_{{i}^{\prime}{j}^{\prime}}^{U}$ is the pairwise interaction energy of the decoy. The frustration index measures how favorable a particular native contact is relative to the set of all other possible contact decoys. Through the *Z*-score, the local energy gap is normalized against the variance of that local energy distribution. If the *Z*-score is significantly large in magnitude, we can be confident that that specific interaction is among the most stable and therefore is unlikely to change during functional motions. If the *Z*-score is small, alternate structures become possible locally. The *Z*-score for the global energy of a protein is scaled to the ratio of the folding to the glass formation temperature. The frustration index indicates the importance of contact informing the funneled energy landscape. As discussed in Ferreiro et al.^4^, the individual contacts can be roughly classified as being either minimally frustrated, highly frustrated, or neutrally frustrated with regard to their frustration level.

### Probing frustration profiles for proteins using the atomistic model Rosetta

To compute localized frustration indices, we use an all-atom force field, the Rosetta energy function^24^. This energy function has been successful at predicting and designing proteins. This energy function was previously adopted by Chen et al.^11^ to localize frustration in their study where the decoys were obtained by sampling a sufficient number of trajectories from molecular dynamics simulations. To achieve greater efficiency here, the decoy ensemble of residue contacts is obtained by randomizing both the residue identities and locations of the residues in contact as was done previously using the AWSEM coarse-grained energy function. This method of decoy generation significantly speeds up the calculations relative to using molecular dynamic sampling. To randomize both the residue identities and residue locations of contacts, we randomly shuffle the protein sequence and then repack the resulting sequence onto the backbone that is provided without perturbing the backbone coordinates within each protein chain to make sure that only the side chains are re-packed. A short Monte-Carlo relaxation is then performed to better eliminate many of the possible side-chain clashes with the backbone fixed. Following this, all the contact energies will be counted into the decoy ensemble (${E}_{{i}^{\prime}{j}^{\prime}}^{U}$). The contact energies of native sequence (${E}_{{i}^{\prime}{j}^{\prime}}^{0}$) are obtained in a similar fashion by omitting the shuffling step. Protein contacts are defined by the *C*_*α*_*C*_*α*_ distances between residues, and a cutoff of 10 Å is used. Since the fully atomistic force field is a many-body construct, the pairwise energy change assigned to forming a contact between residue *i* and *j*, *E*_*ij*_ is defined by considering all the interaction energies that involve changing any of the two residues that are in contact (Eq. (2)).

2 $${E}_{ij\,}={e}_{ij\,}+1/2\mathop{\sum }\limits_{k}^{k\ne j}{e}_{ik\,}+1/2\mathop{\sum }\limits_{l}^{l\ne i}{e}_{jl\,}.$$

In addition to the direct interactions between *i* and *j* (defined as *e*_*ij*_), the addition of the background interactions accounts for the many-body effects elicited by local side-chain reconfigurational changes.

To compute the pairwise energetics (*e*_*ij*_) in the pose that results from relaxation, we employed the REF2015 version of the rosetta energy function^24^, which has a set of well-tested weights for each energy term. The full Rosetta energy function includes many energy terms that are intended to account for the various chemico-physical effects of protein biophysics. Even after relaxation, the repulsive Lennard–Jones interactions used in computing *e*_*ij*_ give rise to very strong fluctuating clashes that can easily be removed by relaxing the backbone. This gives rise to an artificially large variance of the decoy energies. These repulsive clashes would make the algorithm too sensitive for detecting functionally relevant frustration. In the spirit of the van der Waals theory of liquids, it is, however, both easy and appropriate to simply remove the harsh rapidly varying repulsive force term^25^. The atomistic frustration scores in this paper employ this separation of the potential into repulsive and attractive terms to account for small adjustments in structure. Effectively, we expect the harsh repulsive clashes to be removed by further backbone adjustments thus the harsh repulsions simply contribute to changing the configurational entropy^26,27^. They are less sensitive to packing and thus monitor stability more realistically. When the repulsive interactions are explicitly included, the resulting frustration index, which we call the “packing frustration”, turns out to be good at diagnosing the health of a given high-resolution structure (i.e., predicted structure).

### Reporting summary

Further information on research design is available in the Nature Research Reporting Summary linked to this article.

## Supplementary information

Supplementary Information

Reporting Summary

## References

[1] Wolynes PG (2015). Evolution, energy landscapes and the paradoxes of protein folding. Biochimie.

[2] Bryngelson JD, Onuchic JN, Socci ND, Wolynes PG (1995). Funnels, pathways, and the energy landscape of protein folding: a synthesis. Proteins Struct. Funct. Bioinform..

[3] Ferreiro DU, Komives EA, Wolynes PG (2014). Frustration in biomolecules. Q. Rev. Biophys..

[4] Ferreiro DU, Hegler JA, Komives EA, Wolynes PG (2007). Localizing frustration in native proteins and protein assemblies. Proc. Natl Acad. Sci. USA.

[5] Ferreiro DU, Hegler JA, Komives EA, Wolynes PG (2011). On the role of frustration in the energy landscapes of allosteric proteins. Proc. Natl Acad. Sci. USA.

[6] Freiberger MI, Brenda Guzovsky A, Wolynes PG, Gonzalo Parra R, Ferreiro DU (2019). Local frustration around enzyme active sites. Proc. Natl Acad. Sci. USA.

[7] Schafer NP, Kim BL, Zheng W, Wolynes PG (2014). Learning to fold proteins using energy landscape theory. Isr. J. Chem..

[8] Lindorff-Larsen K, Piana S, Dror RO, Shaw DE (2011). How fast-folding proteins fold. Science.

[9] Lammert H, Wolynes PG, Onuchic JN (2012). The role of atomic level steric effects and attractive forces in protein folding. Proteins Struct. Funct. Bioinform..

[10] Lin X (2019). Forging tools for refining predicted protein structures. Proc. Natl Acad. Sci. USA.

[11] Chen J, Schafer NP, Wolynes PG, Clementi C (2019). Localizing frustration in proteins using all-atom energy functions. J. Phys. Chem. B.

[12] Williams, R. J. *Cellular and Molecular Life Sciences.* Vol. 53, 816–829 (Clarendon Press, 1997). 10.1007/s000180050102 PMC11147305 9432285

[13] Wang J (2007). Quantifying intrinsic specificity: a potential complement to affinity in drug screening. Phys. Rev. Lett..

[14] Yan Z, Wang J (2012). Specificity quantification of biomolecular recognition and its implication for drug discovery. Sci. Rep..

[15] Miyashita O, Onuchic JN, Wolynes PG (2003). Nonlinear elasticity, proteinquakes, and the energy landscapes of functional transitions in proteins. Proc. Natl Acad. Sci. USA.

[16] Whitford PC, Onuchic JN, Wolynes PG (2008). Energy landscape along an enzymatic reaction trajectory: hinges or cracks?. HFSP J..

[17] Arnold FH (2015). The nature of chemical innovation: new enzymes by evolution. Q. Rev. Biophys..

[18] Trizac E, Levy Y, Wolynes PG (2010). Capillarity theory for the fly-casting mechanism. Proc. Natl Acad. Sci. USA.

[19] Zheng W, Schafer NP, Davtyan A, Papoian GA, Wolynes PG (2012). Predictive energy landscapes for protein-protein association. Proc. Natl Acad. Sci. USA.

[20] Potoyan DA, Zheng W, Komives EA, Wolynes PG (2016). Molecular stripping in the NF-*κ* B/I*κ* B/DNA genetic regulatory network. Proc. Natl Acad. Sci. USA.

[21] Papoian GA, Ulander J, Wolynes PG (2003). Role of water mediated interactions in protein-protein recognition landscapes. J. Am. Chem. Soc..

[22] Frauenfelder H, Sligar SG, Wolynes PG (1991). The energy landscapes and motions of proteins. Science.

[23] Eisenberg, D. & Kauzmann, W. *The Structure and Properties of Water* (Oxford University Press, 2005).

[24] Alford RF (2017). The Rosetta all-atom energy function for macromolecular modeling and design. J. Chem. Theory Comput..

[25] Chandler D, Weeks JD, Andersen HC (1983). Van der waals picture of liquids, solids, and phase transformations. Science.

[26] Eastwood MP, Hardin C, Luthey-Schulten Z, Wolynes PG (2002). Statistical mechanical refinement of protein structure prediction schemes: cumulant expansion approach. J. Chem. Phys..

[27] Eastwood MP, Hardin C, Luthey-Schulten Z, Wolynes PG (2003). Statistical mechanical refinement of protein structure prediction schemes. II. Mayer cluster expansion approach. J. Chem. Phys..

