# Supplementary material for: Surveying biomolecular frustration at atomic resolution
Source: Nat Commun. 2020 Nov 23;11:5944. doi: 10.1038/s41467-020-19560-9 (PMC7683549; doi:10.1038/s41467-020-19560-9)
Supplement: Supplementary file 1 — Supplementary Information [file 41467_2020_19560_MOESM1_ESM.pdf]

Supplementary Materials for Surveying Biomolecular Frustration at Atomic Resolution

Mingchen Chen[1], Xun Chen[1,2], Nicholas P Schafer[1], Cecilia Clementi[1,2,3,4], Elizabeth A. Komives[6], Diego U. Ferreiro[5], and Peter G. Wolynes*[1,2,3,4]

[1]Center for Theoretical Biological Physics, Rice University, Houston, TX
[2]Department of Chemistry, Rice University, Houston, TX
[3]Department of Physics and Astronomy, Rice University, Houston, TX
[4]Department of Biosciences, Rice University, Houston, TX
[5]Protein Physiology Laboratory, University of Buenos Aires
[6]Department of Chemistry and Biochemistry, University of California at San Diego
*Email: pwolynes@rice.edu, Phone: (713)348-4101

correspondence to: pwolynes@rice.edu

Contents

1 Supplementary Methods **2**
1.1 Cutoff values for classifying frustration levels between amino acids. 2
1.2 Surveying Frustration for Protein-Ligand Complexes. 2
1.3 Probing Frustration Profiles for Proteins Using the Coarse-Grained Model AWSEM. 2
1.4 Protein monomer and complex databases. 3
1.5 The database for allosteric proteins. 3
1.6 The database for enzymes. 3
1.7 The database for protein-ligand complexes. 3
1.8 Pair distribution function to quantify local frustration patterns. 3
1.9 Definition of contact types. 4
1.10 Visualization of protein frustratograms. 4

2 Supplementary Results **5**
2.1 Distribution of localized frustration in natural proteins. 5
2.2 Localized frustration in allosteric proteins. 7
2.3 Localized frustration around catalytic sites in enzymes. 8
2.4 Localized frustration for binding interfaces. 9
2.5 Localizing frustration in protein-ligand complexes. 13
2.6 Waters on wet interfaces of protein-complexes. 14
2.7 Correlation between ligand binding affinity and frustration level. 15
2.8 Checking the convergence effect on pairwise energetics $E^U_{i'j'}$ and $\sigma(E^U_{i'j'})$ from the decoy ensemble. 16
2.9 List of PDBs that were studied for EGFR. 18
2.10 List of selective inhibitors and non-selective inhibitors that were studied. 19
2.11 Example atomistic frustration patterns when there exist multiple pockets on protein surface. . 22

1 Supplementary Methods

1.1 Cutoff values for classifying frustration levels between amino acids.

By using the minimal value and the entropy estimate above for the single pair, a contact between amino acid residues will now be defined as minimally frustrated if its native energy is at the lower end of the distribution of decoy energies, having a Z score of -2.5 or lower magnitude. Conversely, a contact will be defined as highly frustrated if (E_{ij}^0 is at the other end of the distribution with a local frustration index higher than 0.5. If the native energy is in between these limits, we will equivocate and define the contact as neutral.

1.2 Surveying Frustration for Protein-Ligand Complexes.

For protein complexes with non-protein cofactors, we again randomly shuffle the protein sequences across the protein domains with the backbone being fixed, while the precise locations of the ligands/cofactors are allowed to be locally perturbed to accommodate the virtual mutation by adjusting their conformation to the local changes of side-chains inside the pocket. When computing the pairwise energetics (e_{ij}), each cofactor is regarded as a single entity. The contacts between a protein residue and cofactor are defined according to the closest distance between the residue's C_α and any of the heavy atoms inside the cofactor. 10 Angstroms is also used as the cutoff distance. Rosetta, by default, is able to identify the ligands derived from the PDB files directly and processes the ligands with its default parameter sets. In those cases when there is no explicit PDB entries for a given ligand, we process and parameterize the cofactors using the rosetta protocols ($https://www.rosettacommons.org/docs/latest/rosetta_basics/preparation/preparing-ligands$).

For many protein-ligand complexes, the complex structures are not available. We, therefore, model the complex structure by docking the co-factors into the known binding pockets of a protein using Autodock[1]. 2000 docked poses are generated, and the pose with the lowest score is selected for frustration analyses. A contact between amino acid residue and ligand is defined as minimally frustrated if the computed Z score is lower than -1.5, and it will be defined as highly frustrated if the computed Z score is higher than 0.5.

1.3 Probing Frustration Profiles for Proteins Using the Coarse-Grained Model AWSEM.

Frustration analyses of proteins using AWSEM are carried out locally with an executable which is the same as that employed in the frustratometer server[2]. Electrostatic interactions are turned on, and only configurational frustration is calculated to make the appropriate comparison with atomistic frustration. Parra et al provides more details[2].

1.4 Protein monomer and complex databases.

A database of high-quality monomeric protein structures has been generated by Ferreiro et al[3], and was directly adopted in our survey. In brief, 314 monomeric proteins were obtained after filtering out the "membrane", "cell-surface" or small proteins, and excluding structures with low-resolution (worse than 3 Angstrom resolution), chain breaks or co-factors. The list was finally filtered to reduce redundancy at the level of 30 percent sequence similarity.

To study protein-protein interfacial interactions, we used the Benchmark II database based on high-resolution crystal structures from the PDB[4]. This is a database of non-redundant multimeric protein complexes for which most of the individual monomeric crystal structures also are available.

1.5 The database for allosteric proteins.

The database of allosteric proteins was again adopted from Ferreiro et al[5], and 23 pairs of allosteric proteins are included with two experimentally determined structures deposited in the database. The mobile sites are defined as those sites when the deviation of a site between two states is larger than 3 Angstroms after alignment.

1.6 The database for enzymes.

The database of enzymes directly adopts that of Freiberger et al[6], in which the structures of enzymes from the CSA database are downloaded along with the annotated catalytic residues.

1.7 The database for protein-ligand complexes.

The database of protein-ligand complexes was derived from the MOAD database ($http://bindingmoad.org$)[7, 8, 9]. In total, 742 complex structures are obtained after filtering out the small proteins (with fewer than 70 residues), filtering out multimeric proteins, filtering out huge proteins (with larger than 400 residues), and finally filtering out redundancy at the level of 80 percent sequence similarity.

1.8 Pair distribution function to quantify local frustration patterns.

Since frustration is assigned to the contact interactions between two residues, to quantify the density of contacts of each frustration type around a catalytic residue, or any residue in general, we first needed to create virtual particles (VPs). These are points in the space that lie between two residues at the center of mass of the interaction. For each protein structure, we obtained the list of contacts for each frustration class and hence calculated a set of virtual particle coordinates. Subsequently, distances of the virtual particles from the Cα from selected residues, catalytic or control, or the centroid atom from the cofactor molecules were calculated.

g(r) plots were adjusted in their axis ranges to enhance visualizations; however, in all cases g(r) values were normalized such that g(20) = 1.

1.9 Definition of contact types.

In AWSEM, the energetics of tertiary contacts depend on the amino-acid identities (λ), densities (ρ) and interaction distances (r_{ij}) of all residues involved[10, 11]. The contacts are grouped into three categories: short range (distance between Cβ below 6.5 Angstroms), long range (between 6.5 and 9.5 Angstroms), and water-mediated (long-range and exposed to solvent). The energies corresponding to those contact types were previously parameterized using an energy landscape optimization scheme for the interactions between the most commonly occurring 20 natural amino acids.

1.10 Visualization of protein frustratograms.

All the visual representations of the proteins were made using the program Pymol. The contacts are drawn between the Cα atoms of each amino acid. For each ligand, the centroid atom, defined by the closest summation of euclidean distances with the rest atoms in the ligand, is used to draw the connections. The cartoon representation of the protein is adopted and the secondary structures are assigned automatically using Pymol. An alternative visualization script is also provided using the program VMD, and the secondary structure assignments are based on the DSSP program. All the computed results in this study are available and will be visualized through a website ($http://atomisticfrustratometer.org$). Currently, all the results can be obtained through github ($https://github.com/Mingchenchen/AtomicFrustratometer\_Results$).

2 Supplementary Results

2.1 Distribution of localized frustration in natural proteins.

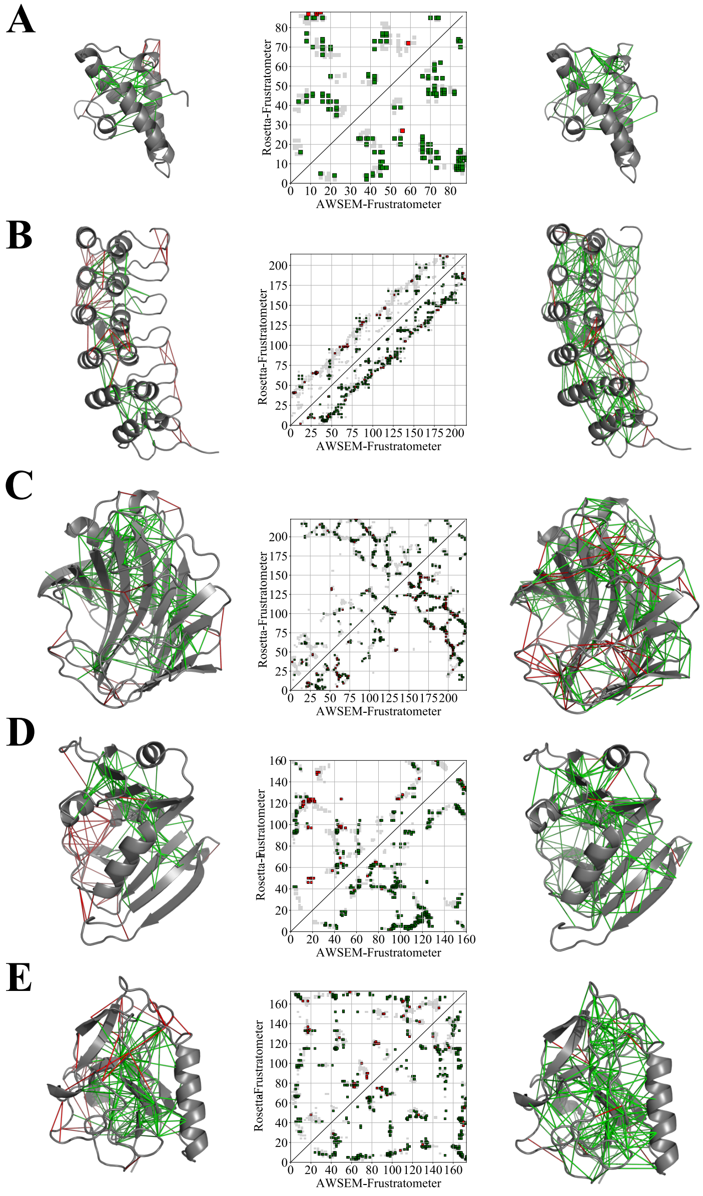

Figure 1: Examples of the localized frustration and minimally frustrated networks in protein structures. The protein backbones are displayed as cartoons. Minimally frustrated interactions and highly frustrated interactions are shown in green and red, respectively; neutral contacts are not drawn. For each protein, the all-atom frustratograms are shown in the left panel, while the coarse-grained frustratometeres are shown in the right panel. Shown in the middle the comparisons between the two different frustratograms are plotted as contact maps, with red squares representing highly frustrated interactions, green representing minimally frustrated contacts, grey for the neutral contacts. (A) Im 7 protein (PDB ID: 7CEI). (B) IκBα (PDB ID: 1NFI). (C) Streptomyces Endoglucanase (PDB ID: 1OA4). (D) Dihydrofolate reductase (PDB ID: 1RX2). (E): Endostatin (PDB ID: 1KOE).

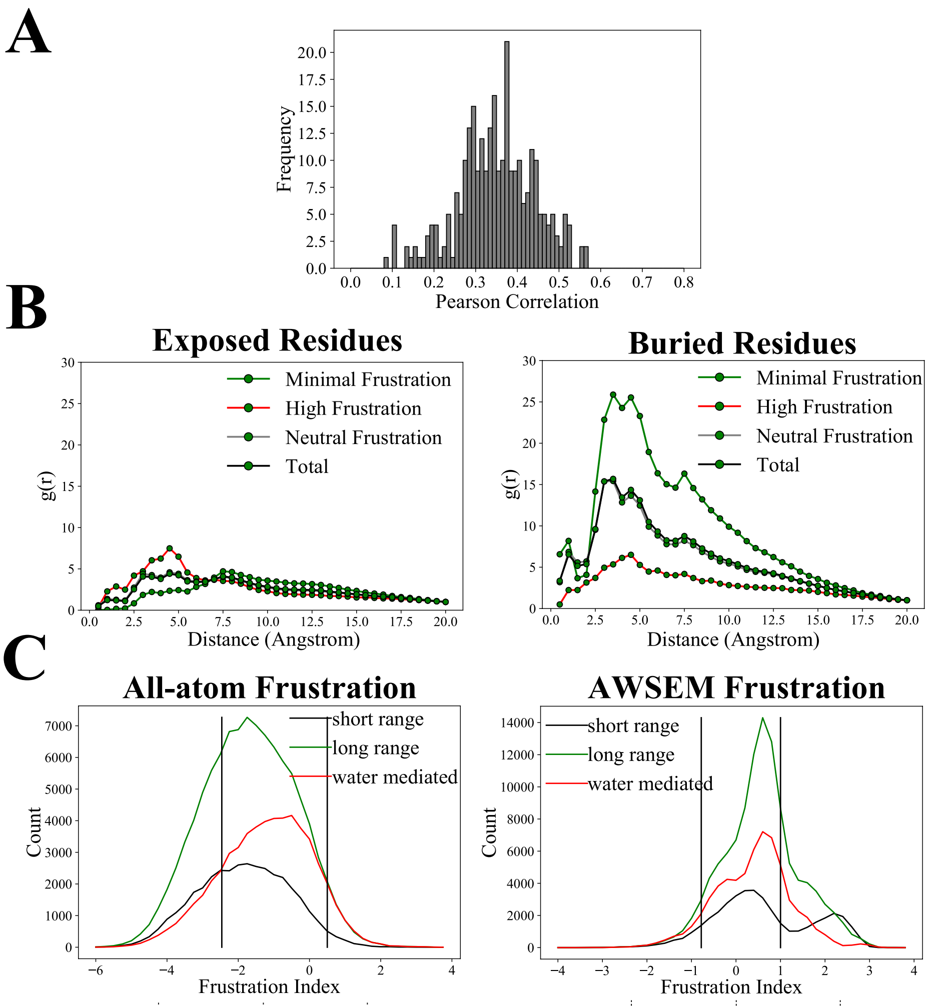

	Minimally Frustrated (%)	Neutrally Frustrated (%)	Highly Frustrated (%)
Short Range	28.5%	67.9%	3.6%
Long Range	24.8%	69.8%	5.4%
Water mediated	15.0%	75.4%	9.7%

	Minimally Frustrated (%)	Neutrally Frustrated (%)	Highly Frustrated (%)
Short Range	34.9%	58.1%	7.0%
Long Range	30.4%	65.3%	4.3%
Water mediated	24.9%	68.4%	6.7%

Figure 2: Statistics of the localized frustration in a database of 314 monomeric protein domains. (A) The distribution of pearson correlation coefficients between all-atom frustration indices and AWSEM frustration indices in each protein is shown in histogram. (B) The pair distribution functions between the centers of mass of contacts classified in groups by their frustration index (minimally frustrated in green, neutral in grey, or highly frustrated in red) to the Cα of either exposed residues (Left panel) or the buried residues (right panel) are shown. (C): Histogram lines showing the distribution of the frustration index in the three different contact classes. The frustration indices were computed for every native contact present in a database of 314 monomeric protein domains. The histogram lines for different contact categories are colored differently (black for short range contacts, green for long range contacts, and red for water mediated contacts). The statistics of minimally frustrated, neutrally frustrated and highly frustrated contacts are counted for each of the three contact categories and shown in the table.

2.2 Localized frustration in allosteric proteins.

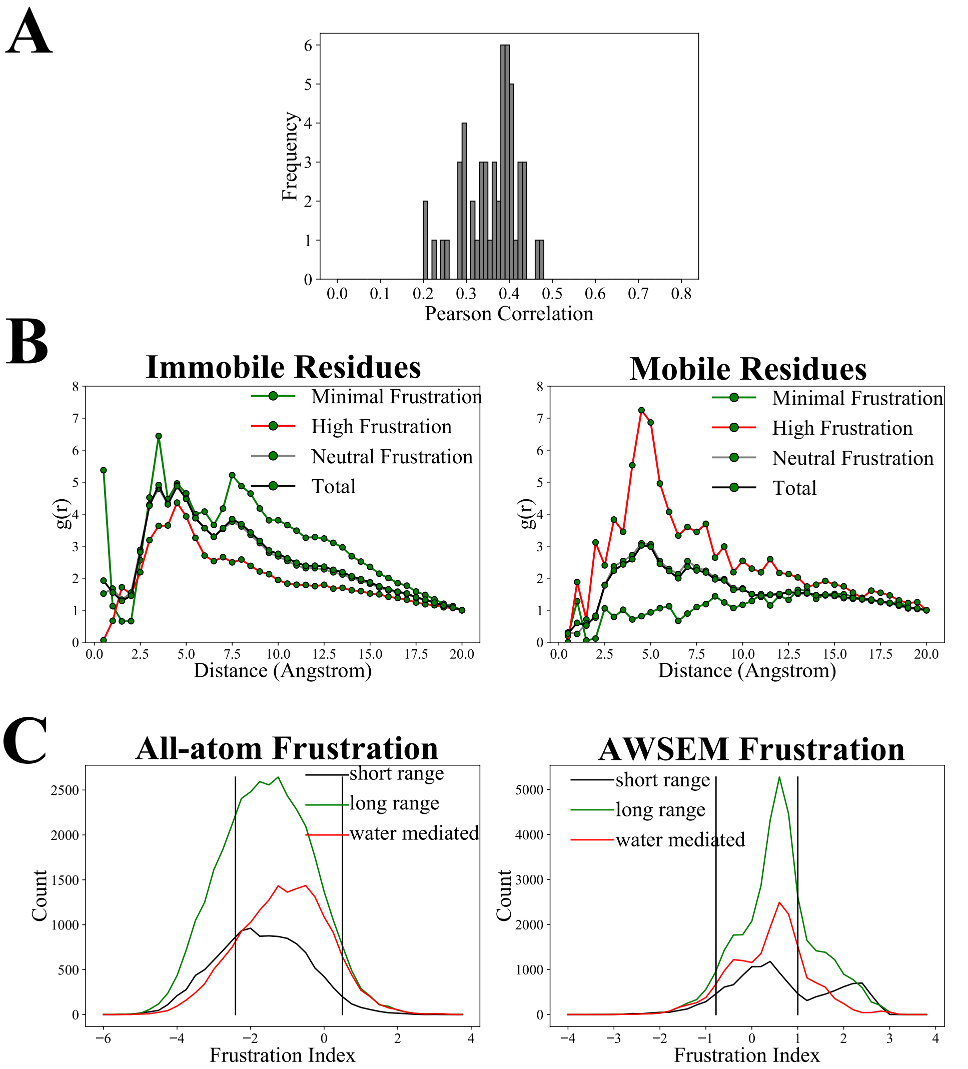

	Minimally Frustrated (%)	Neutrally Frustrated (%)	Highly Frustrated (%)
Short Range	23.9%	71.8%	4.3%
Long Range	20.8%	73.3%	5.9%
Water mediated	11.4%	78.3%	10.3%

	Minimally Frustrated (%)	Neutrally Frustrated (%)	Highly Frustrated (%)
Short Range	36.2%	56.6%	7.2%
Long Range	29.6%	66.3%	4.1%
Water mediated	24.1%	69.6%	6.3%

Figure 3: Statistics of the localized frustration in a database of 23 pairs of allosteric protein domains. (A) The distribution of pearson correlation coefficients between all-atom frustration indices and AWSEM frustration indices in each protein is shown in histogram. (B) The pair distribution functions between the centers of mass of contacts classified by their frustration index (minimally frustrated in green, neutral in grey, or highly frustrated in red) to the Cα of either immobile (Left panel) or the mobile residues (right panel) are shown. (C): Histogram lines showing the distribution of the frustration index in the three different contact classes. The frustration indices were computed for every native contact present in a database of 23 pairs of allosteric protein domains. The histogram lines for different contact categories are colored differently (black for short range contacts, green for long range contacts, and red for water mediated contacts). The statistics of minimally frustrated, neutrally frustrated and highly frustrated contacts are counted for each of the three contact categories and shown in the table.

2.3 Localized frustration around catalytic sites in enzymes.

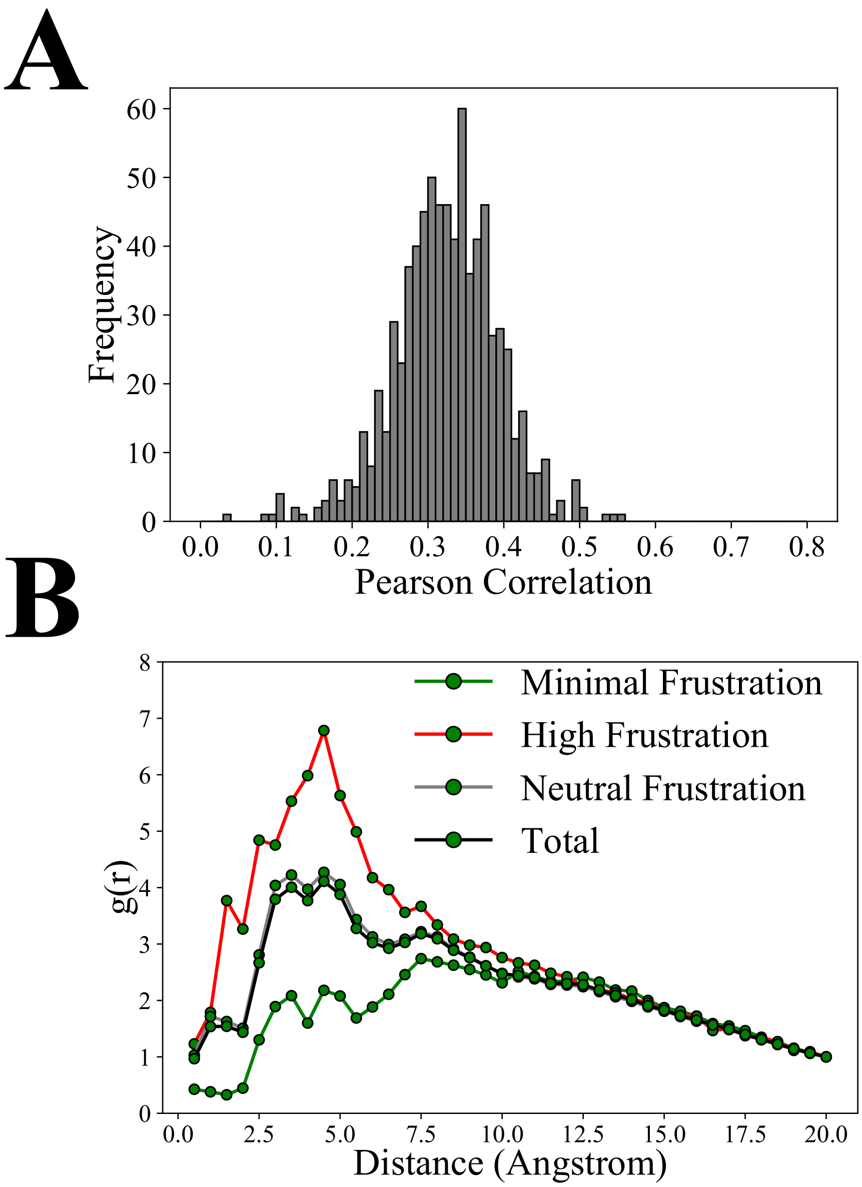

Figure 4: Statistics of the localized frustration around catalytic sites in a database of 891 enzymes. (A) The distribution of pearson correlation coefficients between all-atom frustration indices and AWSEM frustration indices in each protein is shown in histogram. (B) The pair distribution functions between the centers of mass of contacts classified by their frustration index (minimally frustrated in green, neutral in grey, or highly frustrated in red) to the Cα of the catalytic sites are shown.

2.4 Localized frustration for binding interfaces.

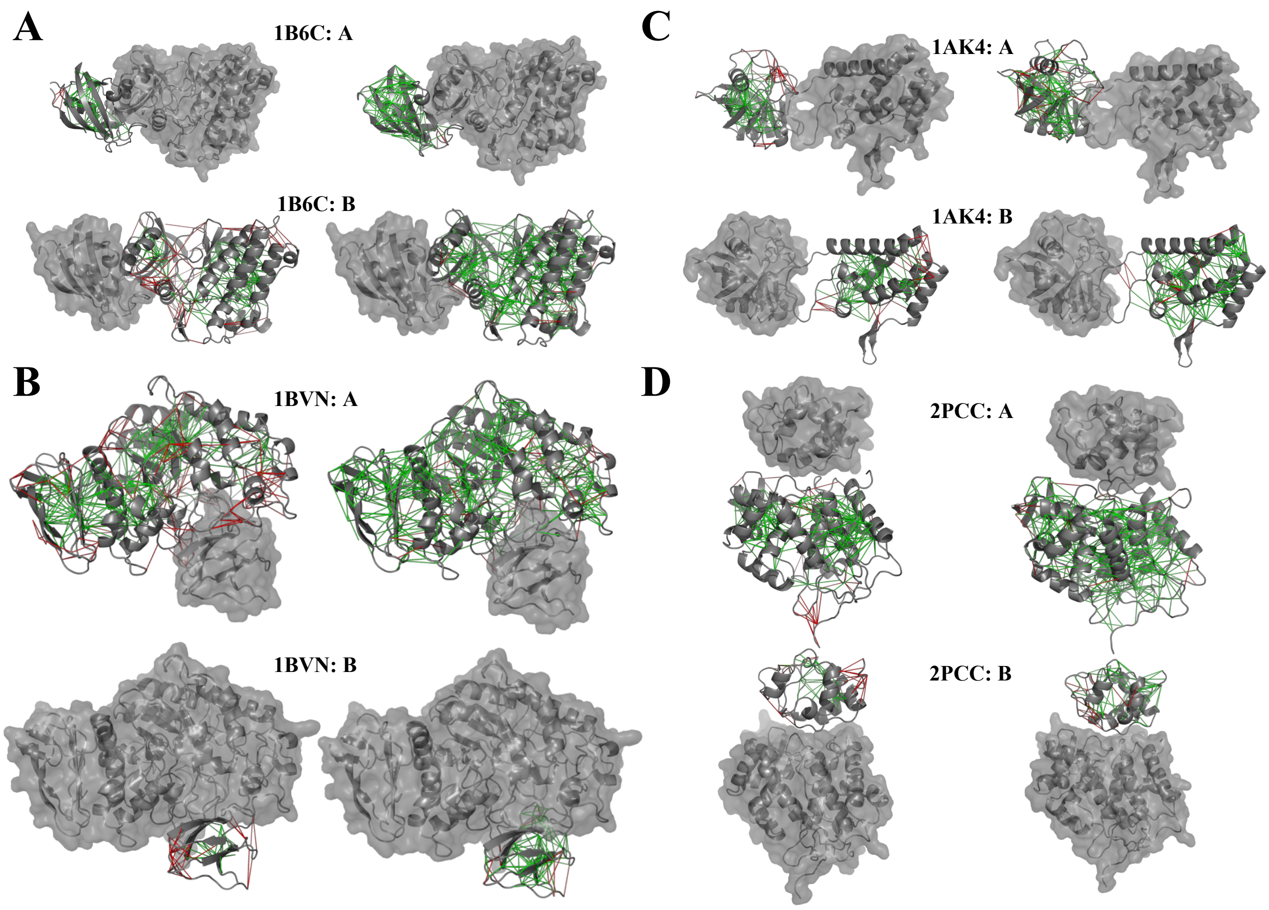

Figure 5: Examples of localized frustration patterns in protein assemblies. The interactions in one monomeric partner are colored according to the contact configurational frustration index, whereas the other partner's surface is colored grey. For each binding complex, the frustration indices are shown as calculated for the unbound monomers using both all-atom frustration (left panel) and coarse-grained frustration (right panel). Complementary views of the same complexes are shown and correspond to the database members which are the cytoplasmic domain of the type 1 TGF-β receptor in complex with fkbp12 (PDB ID: 1B6C) (A), Alpha-amylase bound to tendamistat (PDB ID: 1BVN), cyclophilin bound to the N-terminal domain of HIV-1 capsid (PDB ID: 1AK4) (C) and iso-1-cytochrome C bound to cytochrome C peroxidase (PDB ID: 2PCC) (D). The dinding interfaces in (A) and (B) are largely dry interfaces dominated by non water-mediated contacts, while the interfaces in (C) and (D) are rich in water mediated contacts.

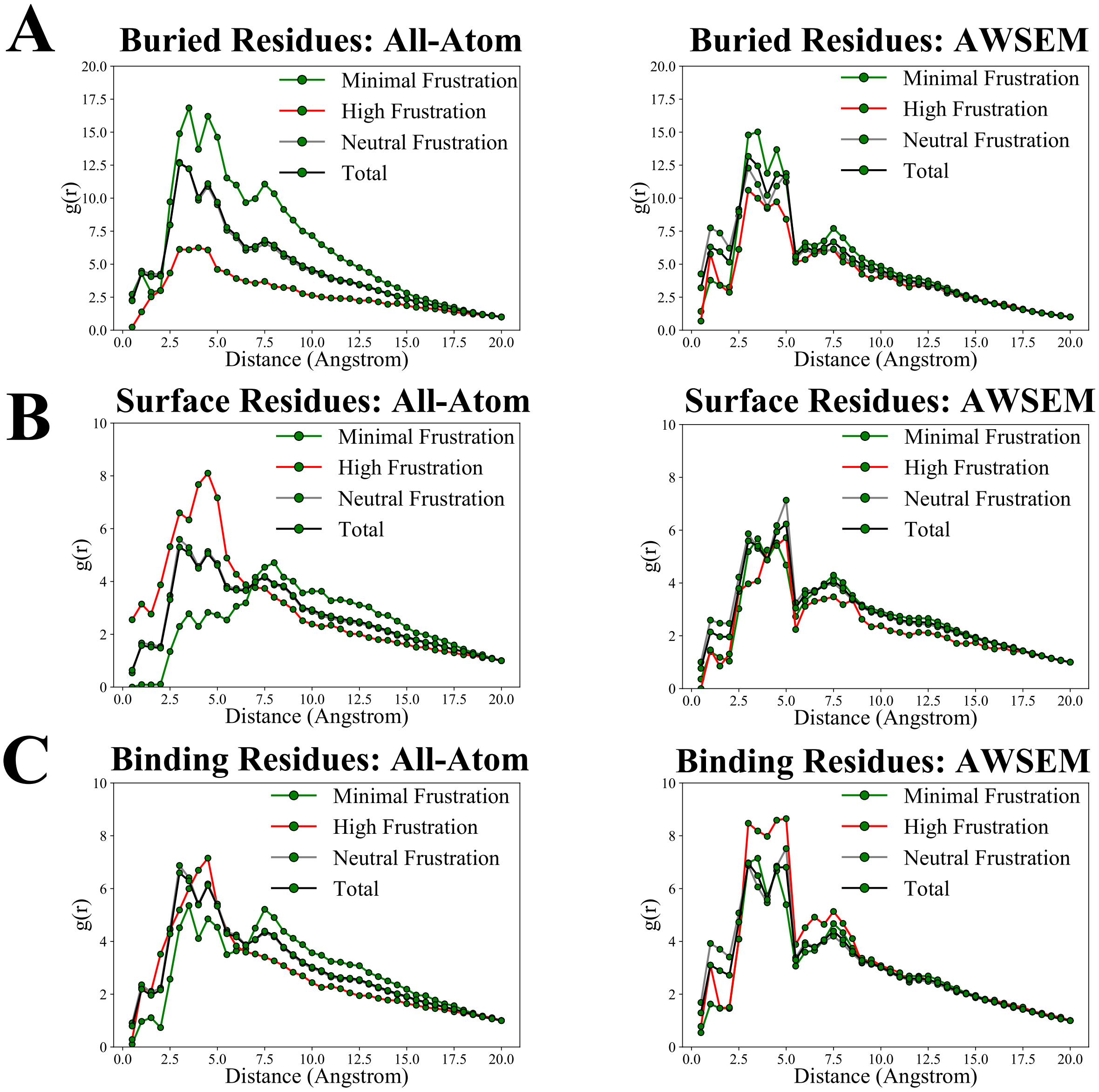

Figure 6: Statistics of the localized frustration around binding sites in a database of 80 protein binding partners. (A) The pair distribution functions between the centers of mass of contacts classified by their frustration index (minimally frustrated in green, neutral in grey, or highly frustrated in red) to the Cα of the buried sites are shown. (B) The pair distribution functions between the centers of mass of contacts classified by their frustration index (minimally frustrated in green, neutral in grey, or highly frustrated in red) to the Cα of the exposed but not involved in binding are shown. (C) The pair distribution functions between the centers of mass of contacts classified by their frustration index (minimally frustrated in green, neutral in grey, or highly frustrated in red) to the Cα of the interface sites are shown.

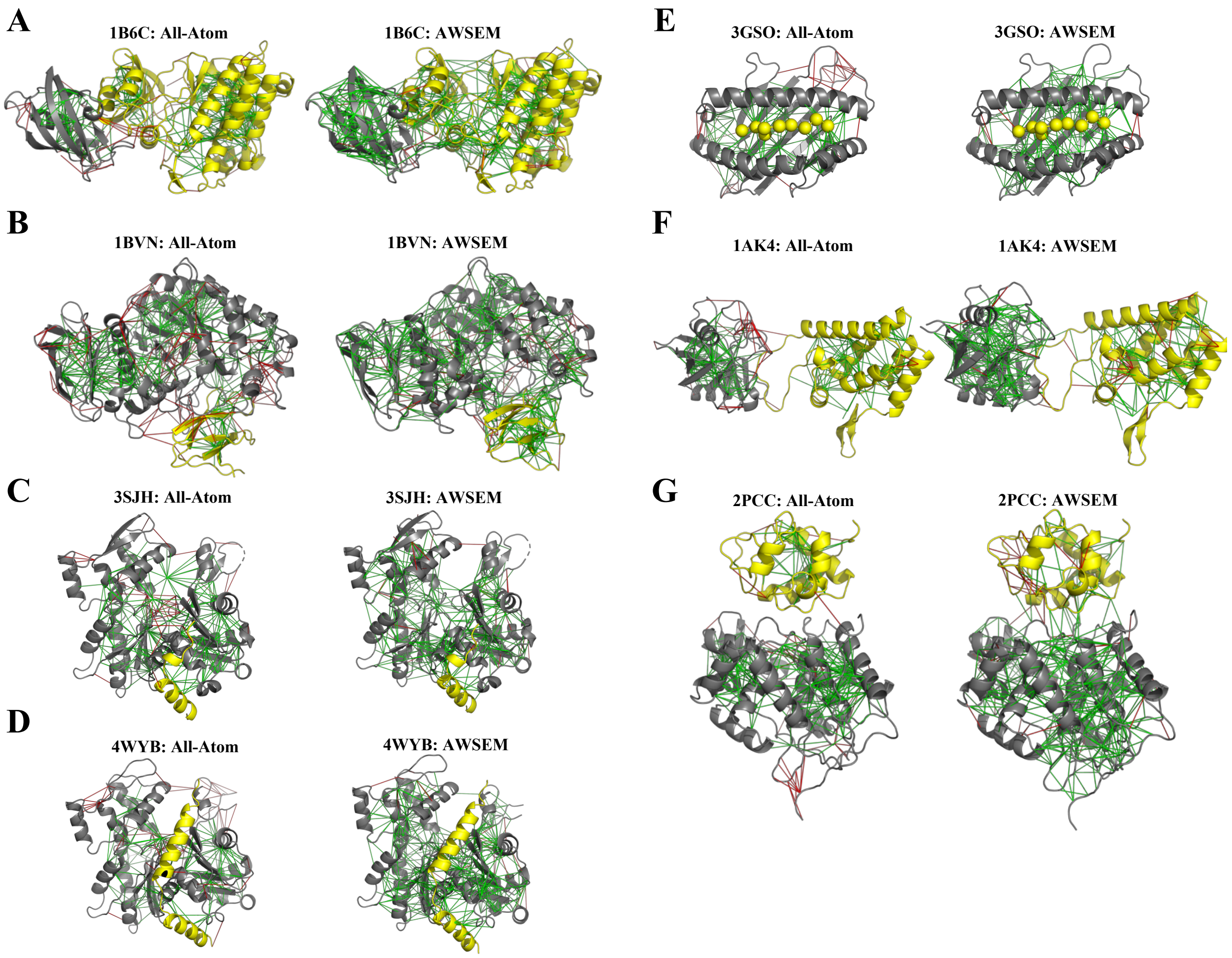

Figure 7: Examples of localized frustration patterns in protein complexes. For each binding complex, the frustration indices are shown as calculated for the complex using both all-atom frustration (left panel) and AWSEM frustration (right panel). Binding interfaces in (A)- (E) are largely dry interfaces dominated by non water-mediated contacts, while the interfaces in (F) and (G) are rich in water mediated contacts.

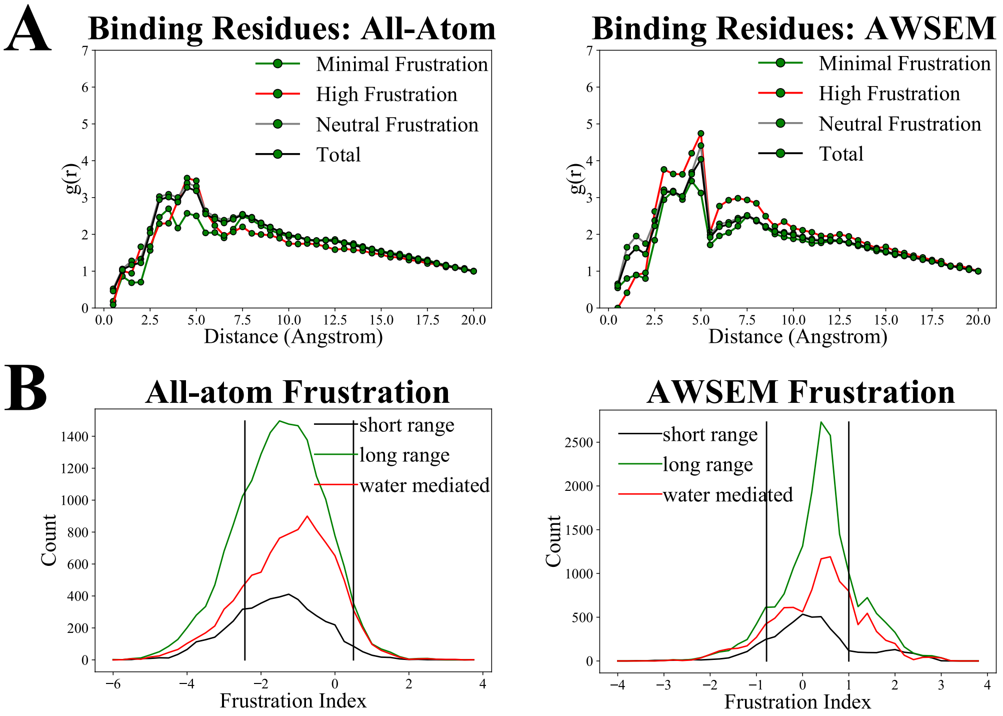

	Minimally Frustrated (%)	Neutrally Frustrated (%)	Highly Frustrated (%)
Short Range	19.6%	76.1%	4.3%
Long Range	17.3%	78.2%	4.6%
Water mediated	14.2%	78.6%	7.2%

	Minimally Frustrated (%)	Neutrally Frustrated (%)	Highly Frustrated (%)
Short Range	19.0%	70.3%	10.7%
Long Range	22.2%	70.8%	7.0%
Water mediated	26.0%	64.4%	9.6%

Figure 8: Statistics of the localized frustration around binding sites in a database of 80 protein assemblies. (A) The pair distribution functions between the centers of mass of contacts classified by their frustration index (minimally frustrated in green, neutral in grey, or highly frustrated in red) to the Cα of the binding sites are shown. (C): Histogram lines showing the distribution of the frustration index involved in binding in the three different contact classes considered. The frustration indices were computed for every native contact present in a database of 80 protein complexes. The histogram lines for different contact categories are colored differently (black for short range contacts, green for long range contacts, and red for water mediated contacts). The statistics of minimally frustrated, neutrally frustrated and highly frustrated contacts are counted for each of the three contact categories and shown in the table.

2.5 Localizing frustration in protein-ligand complexes.

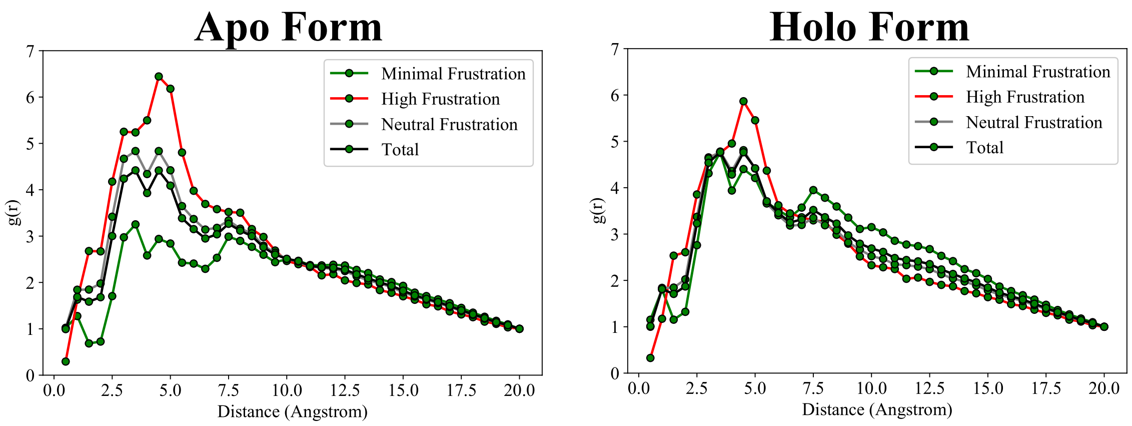

Figure 9: Statistics of the localized frustration around catalytic sites in a database of more than 700 protein-ligand complexes. (A) The pair distribution functions between the centers of mass of contacts classified by their frustration index (minimally frustrated in green, neutral in grey, or highly frustrated in red) to the Cα of the binding sites are shown.

2.6 Waters on wet interfaces of protein-complexes.

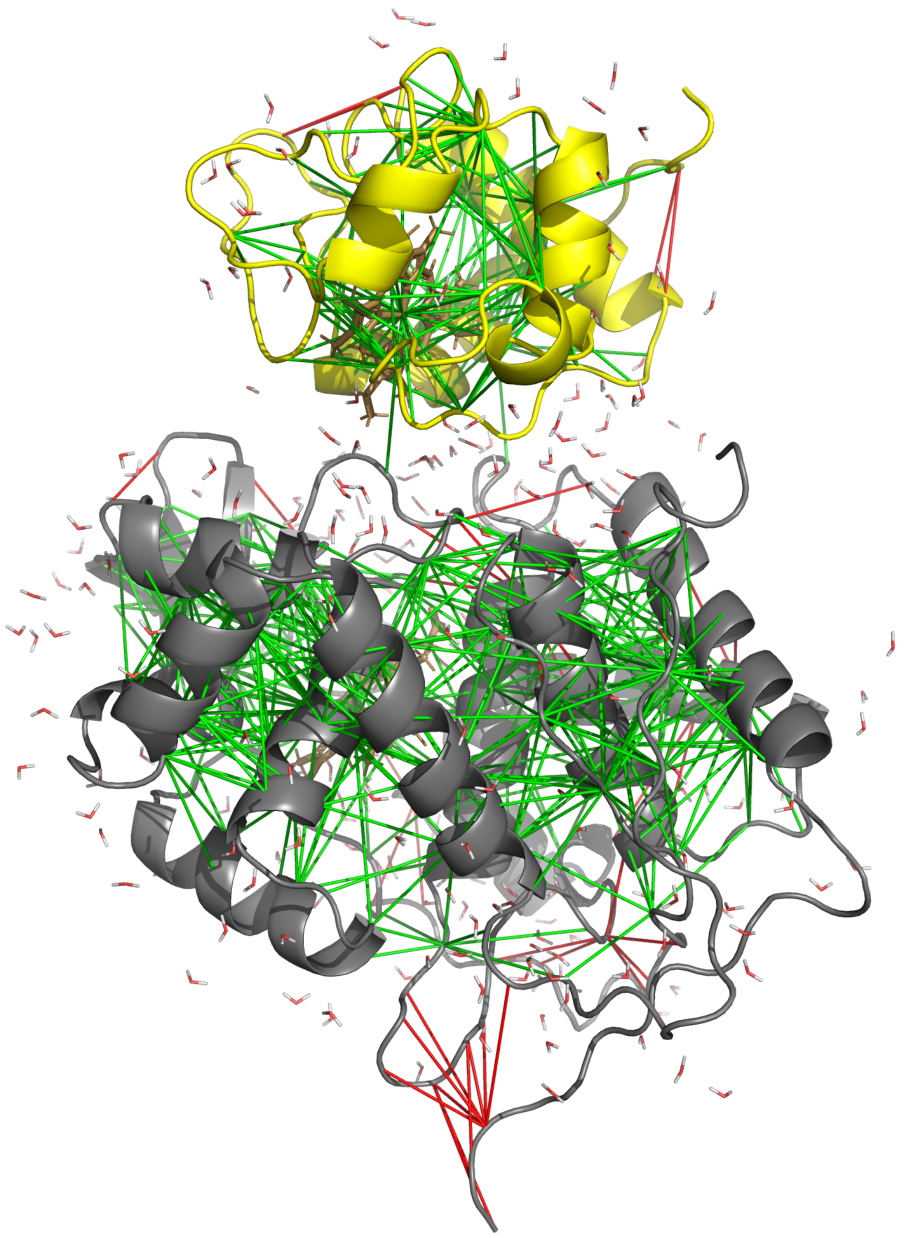

Figure 10: An all-atom frustratogram of 2PCC is shown along with the location of waters on the interface between the partners. Different from Figure 4G which doesn't explicitly considers the ligand during calculating atomic resolution frustration, the ligands are considered in this set of calculations. Most of the water-mediated interactions across the interface are not minimally frustrated according to the Rosetta forcefield.

2.7 Correlation between ligand binding affinity and frustration level.

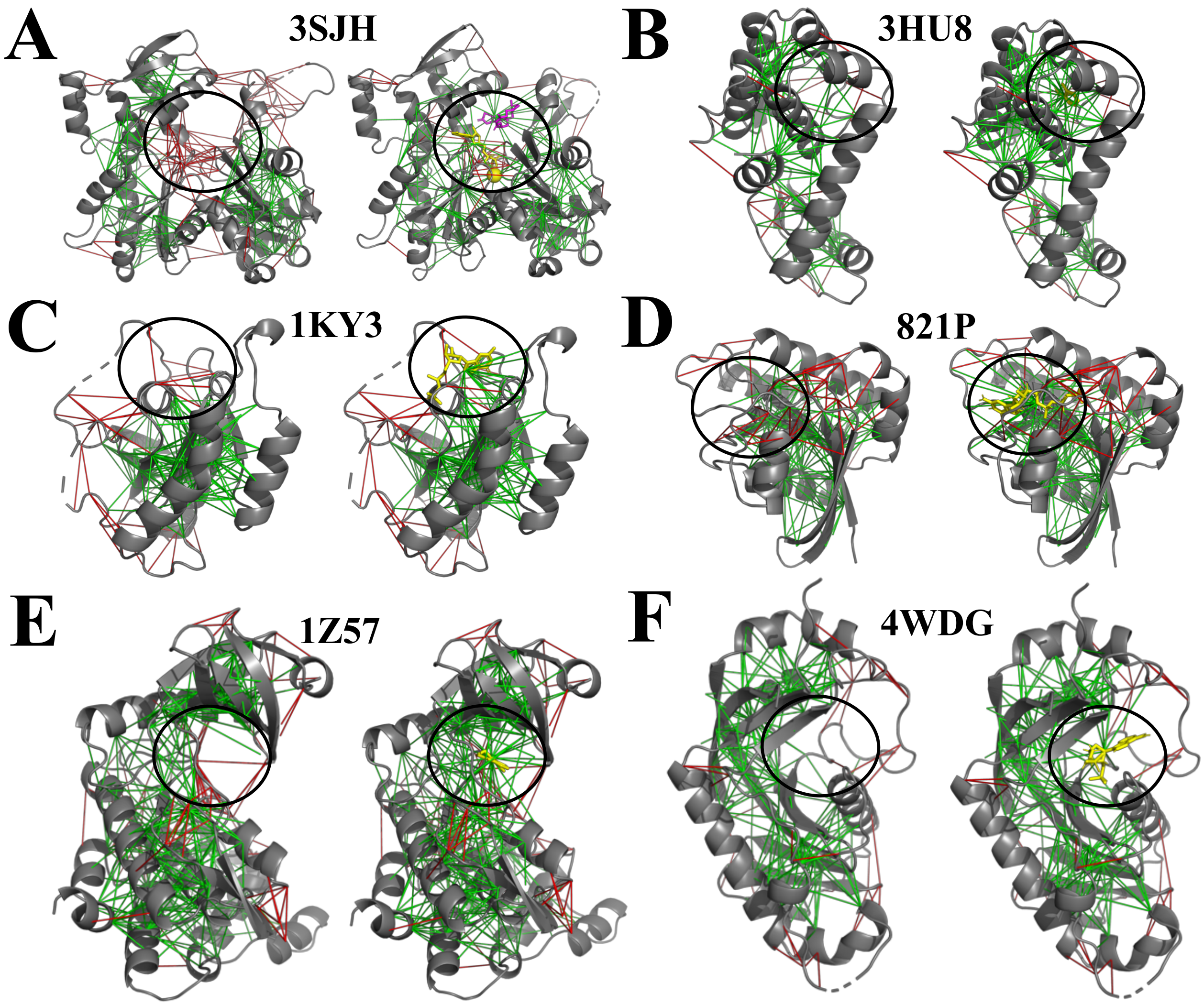

Figure 11: Examples of localized frustration patterns in protein-ligand complexes. For each binding complex, the frustration indices are shown as calculated for the apo form (left panel) and the holo form (right panel).

2.8 Checking the convergence effect on pairwise energetics $E^U_{i'j'}$ and $\sigma(E^U_{i'j'})$ from the decoy ensemble.

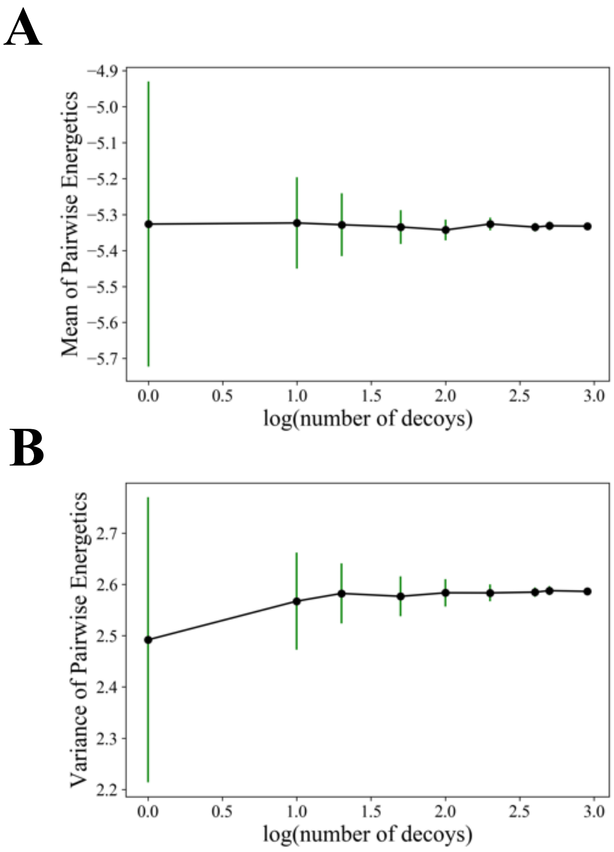

Figure 12: The statistics of pairwise energetics between amino acid residues in an example protein (PDB ID: 1MBA) is obtained through 50 different runs for a fixed size of the decoy ensemble. (A) For each fixed size of decoys, the average value of $E^U_{i'j'}$ and its corresponding error bar over the 50 replicas is plotted. (B) For each fixed size of decoys, the average value of $\sigma(E^U_{i'j'})$ and its corresponding error bar over the 50 replicas is plotted. Generally, the pairwise energetics converges well when the number of decoys is above 200.

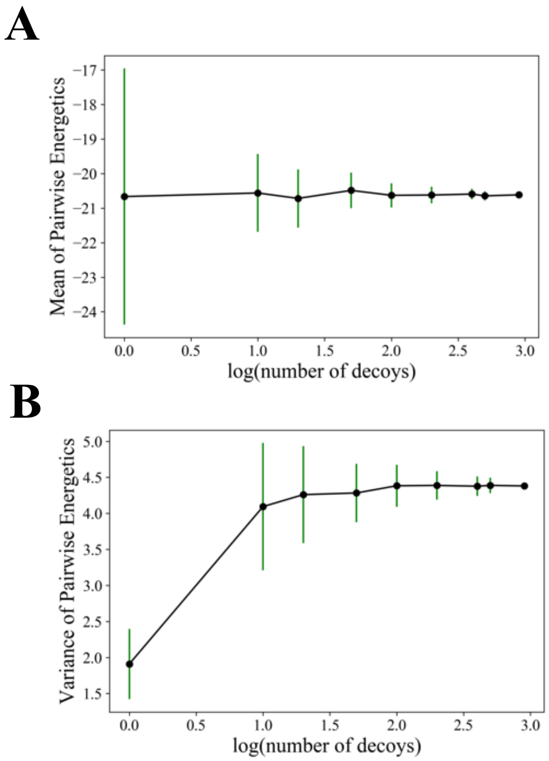

Figure 13: The statistics of pairwise energetics between amino acids and ligand in an example protein (PDB ID: 1MBA) is obtained through 50 different runs for a fixed size of the decoy ensemble. (A) For each fixed size of decoys, the average value of $E^U_{i'j'}$ and its corresponding error bar over the 50 replicas is plotted. (B) For each fixed size of decoys, the average value of $\sigma(E^U_{i'j'})$ and its corresponding error bar over the 50 replicas is plotted. Generally, the pairwise energetics converges well when the number of decoys is above 400.

2.9 List of PDBs that were studied for EGFR.

PDBID	# of minimally frustrated contacts with EGFR	# highly frustrated contacts with EGFR	Affinity (pM)
1xkk	21	0	3
2ito	5	1	123.6
2itp	10	1	11.3
2itt	10	1	1.7
2ity	6	0	53.5
2itz	7	0	2.6
2j6m	12	0	10.9
2jiu	13	0	27.6
2rgp	22	0	30
3bel	22	0	14
3ika	14	0	14
3poz	15	0	23
3w2o	4	0	8400
3w2p	6	0	8100
3w2q	14	0	66
3w2r	12	0	19
3w2s	13	0	6.9
3w32	7	0	75
3w33	23	0	36
4jq7	16	0	393
4jq8	16	0	8
4jr3	13	0	218
4jrv	14	0	29
4li5	10	0	550
4rj4	11	0	16
4rj5	9	0	66
4rj6	10	0	76
4rj7	13	0	22
5c8k	11	0	20
5c8m	11	0	64
5c8n	14	0	28
5cal	20	0	22
5can	13	0	14
5cao	12	0	38
5cap	14	0	52
5caq	17	0	2.7
5cas	16	0	1.4
5cau	15	0	1.6
5cav	7	0	216
5edq	5	0	2
5edr	9	0	34.3
5em5	8	1	262
5em6	16	0	4
5em7	15	0	19
5em8	4	0	1090
5gmp	16	0	0.8
5gnk	16	0	5.3
5gty	19	0	19.01
5hcx	18	0	4.1
5hcy	10	0	1.2
5hg7	12	0	4
5hg8	7	0	3
5hg9	15	0	35
5j9y	17	0	58
5j9z	16	0	16
5ug8	15	0	2
5ug9	15	0	8
5uga	13	0	161
5ugb	5	2	161
5ugc	10	0	13
5yu9	13	0	180

Figure 14: List of PDBs that were studied for EGFR.

2.10 List of selective inhibitors and non-selective inhibitors that were studied.

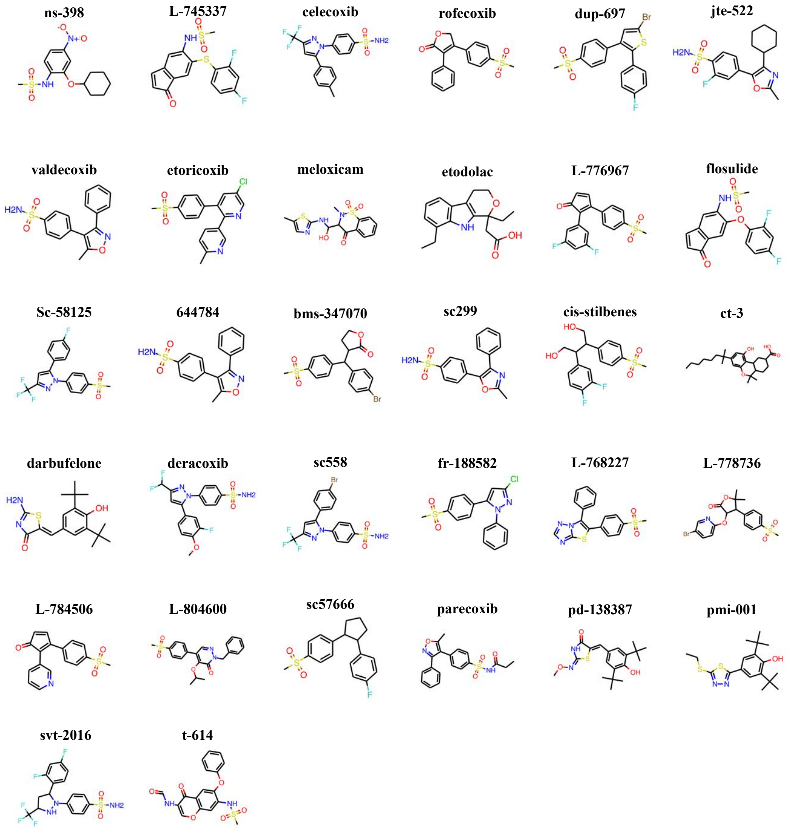

Figure 15: List of selective inhibitors for COX-2.

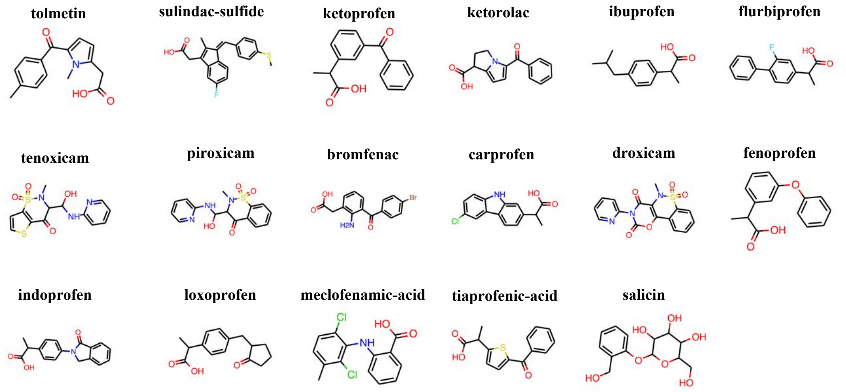

Figure 16: List of non-selective inhibitors for COX-2.

Name	Inhibitor Type	# of minimally frustrated contacts with COX2	# of highly frustrated contacts with COX2	# of minimally frustrated contacts with COX1	# of highly frustrated contacts with COX1	Energy (COX1) in Rosetta Energy Unit	Energy (COX2) in Rosetta Energy Unit
bromfenac	Non-Selective	13	0	13	0	-7.1055	-8.2750
carprofen	Non-Selective	18	0	11	0	-7.0895	-8.2935
droxicam	Non-Selective	20	0	23	0	-10.2440	-9.5840
fenoprofen	Non-Selective	14	0	18	0	-7.3310	-7.1250
flurbiprofen	Non-Selective	20	0	13	0	-7.8840	-9.6250
ibuprofen	Non-Selective	15	0	21	0	-12.9485	-10.8540
indoprofen	Non-Selective	16	0	21	0	-10.5595	-7.9460
ketoprofen	Non-Selective	18	0	15	0	-8.6645	-8.3205
ketorolac	Non-Selective	15	0	16	0	-8.6815	-6.0025
loxoprofen	Non-Selective	13	0	22	0	-8.7025	-12.3915
meclofenamic-acid	Non-Selective	11	1	20	0	-8.1935	-11.8870
piroxicam	Non-Selective	12	0	18	0	-9.2130	-8.3105
salicin	Non-Selective	10	0	13	0	-7.0020	-5.9545
sulindac-sulfide	Non-Selective	23	0	20	0	-12.6930	-13.0430
tenoxicam	Non-Selective	15	0	15	0	-8.9080	-9.9985
tiaprofenic-acid	Non-Selective	19	0	17	0	-8.7155	-9.1255
tolmetin	Non-Selective	16	0	13	0	-11.2395	-8.9175
644784	Selective	22	0	14	0	-9.8660	-10.1280
bms-347070	Selective	13	0	6	0	-7.6155	-10.6695
celecoxib	Selective	19	0	11	0	-11.7145	-10.6550
cis-stilbenes	Selective	19	0	10	0	-9.1205	-11.2085
ct-3	Selective	22	0	9	0	-15.8995	-18.7490
darbufelone	Selective	14	0	20	0	-15.6355	-16.6055
deracoxib	Selective	12	0	11	0	-9.7825	-7.8935
dup-697	Selective	24	0	19	0	-10.6335	-12.1555
etodolac	Selective	19	0	17	0	-11.5945	-10.6360
etoricoxib	Selective	24	0	22	0	-12.9600	-13.1700
flosulide	Selective	16	0	16	0	-9.4670	-8.2070
fr-188582	Selective	19	0	18	0	-10.7910	-11.2590
jte-522	Selective	24	0	18	0	-10.5960	-14.1875
l-745337	Selective	17	0	21	0	-11.5750	-12.7080
l-758115	Selective	15	0	7	0	-9.6975	-13.2595
l-768277	Selective	19	0	10	0	-10.4490	-11.8355
l-776967	Selective	20	0	17	0	-9.6640	-10.7305
l-778736	Selective	13	0	16	0	-11.8430	-14.1935
l-784506	Selective	14	0	12	0	-10.0525	-8.8250
l-804600	Selective	19	0	13	0	-14.2120	-12.9905
meloxicam	Selective	25	0	18	0	-10.8400	-12.8630
ns-398	Selective	11	0	13	0	-8.6955	-13.9165
parecoxib	Selective	8	0	13	0	-12.7405	-12.0120
pd-138387	Selective	24	0	21	0	-20.7925	-21.3585
pmi-001	Selective	19	0	4	0	-16.0320	-19.8220
rofecoxib	Selective	8	0	14	0	-9.9355	-10.3945
sc-58125	Selective	20	0	16	0	-9.1870	-9.8275
sc299	Selective	19	0	14	0	-12.6050	-7.9170
sc558	Selective	19	0	18	0	-8.4745	-6.2100
sc57666	Selective	14	1	17	0	-10.4975	-9.1860
svt-2016	Selective	13	0	15	0	-5.7315	-8.4250
t-614	Selective	13	0	17	0	-10.6870	-11.0805
valdecoxib	Selective	16	0	8	0	-9.6685	-9.9705

Figure 17: Summary of frustration patterns of both non-selective and selective inhibitors.

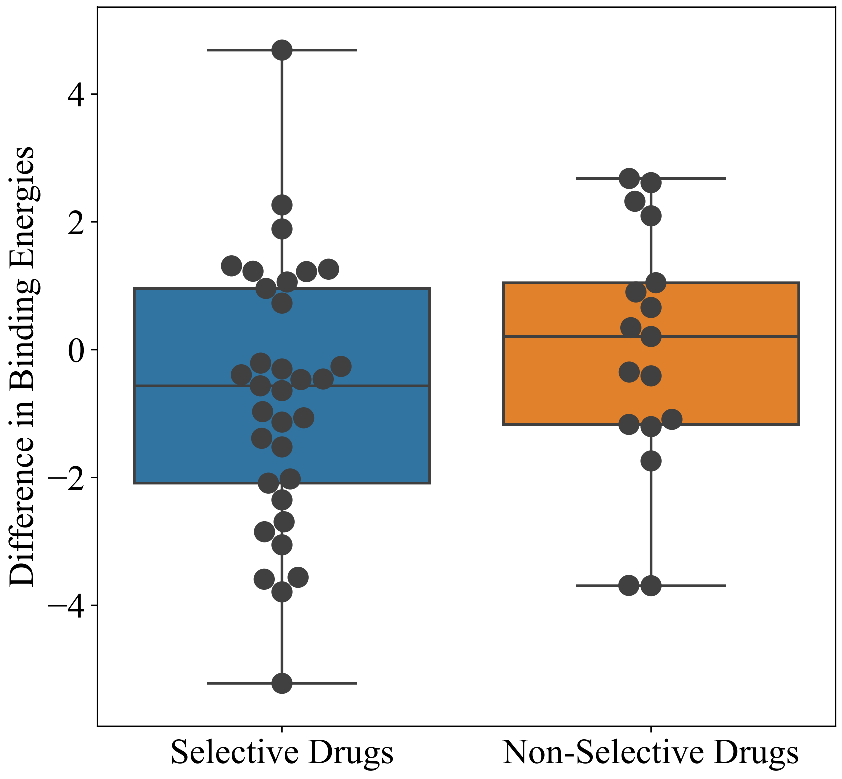

Figure 18: Box plot of the difference in binding energy of COX2 over COX1 is shown for both the selective drugs of COX2 and non-selective drugs.

2.11 Example atomistic frustration patterns when there exist multiple pockets on protein surface.

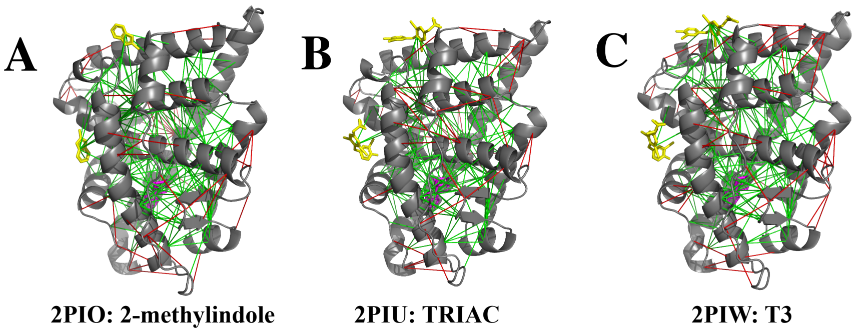

Figure 19: Atomistic frustratograms of androgen receptors in complex with ligands on two binding pockets. Crystal structure of the ligand binding domain (LBD) of androgen receptor is shown in complex with the small molecule modulators (PDB ID: 2PIO, 2PIU and 2PIW)[12]. There are two binding pockets identified on the surface: the activation function 2 (AF-2, pocket on the left) and the binding function (BF-3, on the top). The three types of ligands (shown in yellow sticks) bind to the two pockets at the same time. In general, 2-methylindole (2MI) share similar frustration level in both pockets, while TRIAC and T3 is more minimally frustrated at the BF-3 pocket, corresponding well with the observation that TRIAC interacts preferentially at BF-3 vs. AF-2[12].

Supplementary References

[1] G. M. Morris, R. Huey, W. Lindstrom, M. F. Sanner, R. K. Belew, D. S. Goodsell, and A. J. Olson, "AutoDock4 and AutoDockTools4: Automated docking with selective receptor flexibility," vol. 30, no. 16, pp. 2785–2791.

[2] R. G. Parra, N. P. Schafer, L. G. Radusky, M.-Y. Tsai, A. B. Guzovsky, P. G. Wolynes, and D. U. Ferreiro, "Protein frustratometer 2: a tool to localize energetic frustration in protein molecules, now with electrostatics," vol. 44, pp. W356–360.

[3] D. U. Ferreiro, J. A. Hegler, E. A. Komives, and P. G. Wolynes, "Localizing frustration in native proteins and protein assemblies," vol. 104, no. 50, pp. 19819–19824.

[4] J. Mintseris, K. Wiehe, B. Pierce, R. Anderson, R. Chen, J. Janin, and Z. Weng, "Protein-protein docking benchmark 2.0: An update," vol. 60, no. 2, pp. 214–216.

[5] D. U. Ferreiro, J. A. Hegler, E. A. Komives, and P. G. Wolynes, "On the role of frustration in the energy landscapes of allosteric proteins," vol. 108, no. 9, pp. 3499–3503.

[6] M. I. Freiberger, A. B. Guzovsky, P. G. Wolynes, R. G. Parra, and D. U. Ferreiro, "Local frustration around enzyme active sites," vol. 116, no. 10, pp. 4037–4043.

[7] L. Hu, M. L. Benson, R. D. Smith, M. G. Lerner, and H. A. Carlson, "Binding MOAD (mother of all databases)," vol. 60, no. 3, pp. 333–340.

[8] A. Ahmed, R. D. Smith, J. J. Clark, J. B. Dunbar, and H. A. Carlson, "Recent improvements to binding MOAD: a resource for proteinligand binding affinities and structures," vol. 43, pp. D465–D469.

[9] R. D. Smith, J. J. Clark, A. Ahmed, Z. J. Orban, J. B. Dunbar, and H. A. Carlson, "Updates to binding MOAD (mother of all databases): Polypharmacology tools and their utility in drug repurposing," vol. 431, no. 13, pp. 2423–2433.

[10] G. A. Papoian, J. Ulander, M. P. Eastwood, Z. Luthey-Schulten, and P. G. Wolynes, "Water in protein structure prediction," vol. 101, no. 10, pp. 3352–3357.

[11] A. Davtyan, N. P. Schafer, W. Zheng, C. Clementi, P. G. Wolynes, and G. A. Papoian, "AWSEM-MD: protein structure prediction using coarse-grained physical potentials and bioinformatically based local structure biasing," vol. 116, no. 29, pp. 8494–8503.

[12] E. Estebanez-Perpina, L. A. Arnold, P. Nguyen, E. D. Rodrigues, E. Mar, R. Bateman, P. Pallai, K. M. Shokat, J. D. Baxter, R. K. Guy, P. Webb, and R. J. Fletterick, "A surface on the androgen receptor that allosterically regulates coactivator binding," vol. 104, no. 41, pp. 16074–16079.
